# Reduced Environmental Stimulation in Anorexia Nervosa: An Early-Phase Clinical Trial

**DOI:** 10.3389/fpsyg.2020.567499

**Published:** 2020-10-06

**Authors:** Sahib S. Khalsa, Scott E. Moseman, Hung-Wen Yeh, Valerie Upshaw, Beth Persac, Eric Breese, Rachel C. Lapidus, Sheridan Chappelle, Martin P. Paulus, Justin S. Feinstein

**Affiliations:** ^1^Laureate Institute for Brain Research (LIBR), Tulsa, OK, United States; ^2^Oxley College of Health Sciences, The University of Tulsa, Tulsa, OK, United States; ^3^Laureate Eating Disorders Program, Tulsa, OK, United States; ^4^Children’s Mercy Health System, Kansas City, MO, United States; ^5^Department of Psychology, The University of Tulsa, Tulsa, OK, United States

**Keywords:** eating disorder, floatation therapy, interoception, stress, body image, body awareness, interoceptive awareness

## Abstract

Reduced Environmental Stimulation Therapy (REST) alters the balance of sensory input to the nervous system by systematically attenuating sensory signals from visual, auditory, thermal, tactile, vestibular, and proprioceptive channels. Previous research from our group has shown that REST *via* floatation acutely reduces anxiety and blood pressure (BP) while simultaneously heightening interoceptive awareness in clinically anxious populations. Anorexia nervosa (AN) is an eating disorder characterized by elevated anxiety, distorted body representation, and abnormal interoception, raising the question of whether REST might positively impact these symptoms. However, this approach has never been studied in eating disorders, and it is unknown whether exposure to floatation REST might worsen AN symptoms. To examine these possibilities, we conducted an open-label study to investigate the safety and tolerability of REST in AN. We also explored the acute impact of REST on BP, affective symptoms, body image disturbance, and interoception. Twenty-one partially weight-restored AN outpatients completed a protocol involving four sequential sessions of REST: reclining in a zero-gravity chair, floating in an open pool, and two sessions of floating in an enclosed pool. All sessions were 90 min, approximately 1 week apart. We measured orthostatic BP before and immediately after each session (primary outcome), in addition to collecting BP readings every 10 min during the session using a wireless waterproof system as a secondary outcome measure. Each participant’s affective state, awareness of interoceptive sensations, and body image were assessed before and after every session (exploratory outcomes). There was no evidence of orthostatic hypotension following floating, and no adverse events (primary outcome). Secondary analyses revealed that REST induced statistically significant reductions in BP (*p* < 0.001; Cohen’s *d*, 0.2–0.5), anxiety (*p* < 0.001; Cohen’s *d*, >1) and negative affect (*p* < 0.01; Cohen’s *d*, >0.5), heightened awareness of cardiorespiratory (*p* < 0.01; Cohen’s *d*, 0.2–0.5) but not gastrointestinal sensations, and reduced body image dissatisfaction (*p* < 0.001; Cohen’s *d*, >0.5). The findings from this initial trial suggest that individuals with AN can safely tolerate the physical effects of REST *via* floatation. Future randomized controlled trials will need to investigate whether these initial observations of improved anxiety, interoception, and body image disturbance occur in acutely ill AN populations.

**Clinical Trial Registration:**

ClinicalTrials.gov; Identifier: NCT02801084 (April 01, 2016).

## Introduction

Anorexia nervosa is an unusually deadly disorder with the highest mortality risk of all psychiatric disorders (Sullivan, 1995; Suokas et al., 2013), carrying an estimated standardized mortality rate two to three times higher than schizophrenia, bipolar disorder, and unipolar depression (Arcelus et al., 2011; Hoang et al., 2014). Disability from AN peaks during the second and third decades of life, jeopardizing developmental milestones including adult individuation and educational and occupational achievements. Although many AN patients die from complications associated with starvation, others die as a result of suicide (Keshaviah et al., 2014). Of those remaining, 20% are chronically ill (Steinhausen, 2002; Bulik et al., 2007; Lock, 2010; McElroy et al., 2015), with relapse rates as high as 30–50% following inpatient treatment (Khalsa et al., 2017; Berends et al., 2018).

Individuals with AN show evidence of heightened anxiety expression, including premorbid anxious personality traits, and the disorder shares a high degree of comorbidity (nearly 60–80%) with anxiety disorders and depression (Bulik et al., 1997; Kaye et al., 2004). The diagnosis of an anxiety disorder has been observed to increase the subsequent risk of developing AN (Meier et al., 2015), suggesting the possibility of shared etiological mechanisms or common pathophysiological pathways. However, medical treatments for anxiety and mood disturbances show limited efficacy in AN, with serotonergic agents being the primary long-term medication treatment option (Himmerich and Treasure, 2018). One especially concerning finding is that anxiolytic medications that are effective at reducing anxiety over the short term in anxiety-disordered patients, such as benzodiazepines, are ineffective in lowering the anxiety associated with AN (Steinglass et al., 2014). Alternative anxiolytic medications, such as beta-blockers, are often contraindicated in these patients due to the compensatory bradycardia that often follows chronic caloric restriction in AN patients. Therefore, additional treatments which can effectively ameliorate affective disturbances in AN are needed.

One pathophysiological model of AN posits that these individuals have a fundamentally disturbed relationship between the way that afferent internal bodily signals are processed in the brain, and that this disturbance causally contributes to the emergence of aversive visceral sensations and emotions when exposed to food or food-related stimuli (Kaye et al., 2009, 2011). For example, individuals with AN show evidence of exaggerated perceptual processing of anxiety-associated cardiac and respiratory sensations (e.g., heightened feelings of palpitations and dyspnea) (Khalsa et al., 2015), as well as abnormal neural activation in interoceptive brain regions such as the cingulate and insular cortices (Kerr et al., 2016; Berner et al., 2017). However, the evidence for causal influences of interoception on the development and expression of AN and other eating disorders is limited by a lack of basic and clinical studies focusing on mechanistic underpinnings (Martin et al., 2019).

Reduced Environmental Stimulation Therapy *via* floatation is a relatively unexplored non-pharmacological intervention that has recently begun to be investigated for potential anxiolytic effects (Jonsson and Kjellgren, 2016; Feinstein et al., 2018a,b). The REST experience is calibrated so that input from exteroceptive sensory channels (e.g., visual, auditory, olfactory, gustatory, thermal, and tactile) is minimized, as is most vestibular, gravitational, and proprioceptive input, movement, and speech. As a first step toward exploring whether floatation-REST could help individuals with anxiety and depression, we conducted an open-label trial in 50 anxious and depressed individuals, spanning a range of different anxiety and stress-related disorders (including posttraumatic stress disorder, generalized anxiety disorder, social anxiety disorder, panic disorder, and agoraphobia) (Feinstein et al., 2018b). A single 1-h session of floatation-REST was well tolerated by the anxious sample, with no major safety concerns or adverse events. Regardless of diagnosis, the float experience induced a strong short-term reduction in state anxiety and a substantial improvement in mood. In a recent follow-up study using a within-subject crossover design, we recruited 31 participants with clinically elevated levels of anxiety to undergo a 90-min session of floatation-REST and an exteroceptive comparison condition (watching a relaxing documentary film) (Feinstein et al., 2018a). Measures of self-reported affect and interoceptive awareness were collected before and after each session, and BP was measured during each session. Floatation-REST generated a significant anxiolytic effect relative to the comparison condition that was characterized by reductions in state anxiety and muscle tension and increases in feelings of relaxation and serenity. In addition, significant BP reductions were evident throughout the float session. Despite the observed anxiolytic effects in these individuals, exposure to the float environment significantly enhanced awareness for interoceptive (cardiac and respiratory) sensations, a finding that we attributed to a process of reciprocal inhibition (Wolpe, 1968).

Based on our initial findings with anxious individuals, we wondered whether REST *via* floatation might positively impact affective and interoceptive symptoms in individuals with AN. However, there have been no studies documenting the safety or tolerability of the procedure in eating disorders populations (acutely ill or remitted/recovered), and we could find only one brief theoretical review of the topic suggesting that “there are some qualities of REST that make it particularly appropriate for the treatment of eating disorders” (Barabasz, 1993). Moreover, we were uncertain whether exposure to REST might worsen AN symptoms. We therefore aimed to conduct an open-label clinical trial to assess the safety and tolerability of REST in AN, and secondarily, to explore the impact of this procedure on clinically relevant symptoms related to affective experience, body image disturbance, and interoception. With no previous studies of REST in eating disorder populations, we focused our recruitment for this initial study on partially weight-restored AN individuals drawn from outpatient settings (1) in case the intervention was anxiety provoking or non-therapeutic and (2) preclude imminent fall risk under normal physical activity levels (as can occur in AN individuals who are severely underweight).

The primary objective of the study was to investigate whether there is evidence of negative health consequences associated with exposure to the REST environment in partially weight-restored AN. We selected orthostatic hypotension as our primary outcome for safety since (1) our prior research (Feinstein et al., 2018a) identified reductions in BP as an acute effect associated with floating, (2) orthostatic hypotension could increase fall risk when transitioning from laying to standing (an action occurring at the end of each float) (Sachs et al., 2016), and (3) orthostatic hypotension is a major medical condition associated with acute dehydration that is especially common in underweight and even partially weight-restored AN (Lanier et al., 2011). We hypothesized that floating would be safe and well tolerated by individuals with AN and predicted that there would be no adverse physical effects (e.g., no orthostatic BP reductions, or falls, upon standing). We chose several secondary outcome measures including anxiety, stress, mood, body representation, and interoceptive awareness (introduced further in the next section) and investigated the effects of floating on these subjective measures using two-tailed hypothesis tests and Cohen’s *d* effect size.

## Materials and Methods

### Aim

The primary objective of this study was to determine whether individuals with partially weight-restored AN would exhibit evidence of orthostatic hypotension following REST. We defined orthostatic hypotension (primary outcome) as a drop of ≥20 mmHg in SBP or a drop of ≥10 mmHg in DBP when measured shortly after transitioning from lying down to standing, according to consensus guidelines (Kaufmann, 1996). The secondary objective of this study was to examine the acute effects of REST on BP during floating and subjective measures of emotional experience (including anxiety and mood), body image disturbance, and interoception. These secondary aims were exploratory and intended to provide information on the subjective changes induced by REST in individuals with AN, assisting in the identification of potentially useful targets for future studies.

### Participant Recruitment

Participants were recruited *via* online and print advertisements and *via* referral from eating disorder treatment providers in the local community. To be included in the study, participants were required to have met the *Diagnostic and Statistical Manual 5* (DSM-5) criteria for a lifetime diagnosis of AN during an interview with a board-certified psychiatrist. Additionally, all participants were required to be partially weight restored to a BMI range above the cut-off for a current diagnosis of AN according to ICD10 (World Health Organization, 1993), defined as having a BMI of >17.5. Exclusion criteria included the presence of any schizophrenia spectrum disorder, other psychotic disorder, and bipolar and related disorders. Inpatients were excluded, as were individuals reporting active suicidal ideation with intent or plan (as determined by psychiatric interview). Subjects who exhibited orthostatic hypotension prior to REST (defined as a drop of ≥20 mmHg in SBP or a drop of ≥10 mmHg in DBP when measured shortly after transitioning from lying down to standing) were excluded. We chose to examine BP changes between lying and standing, as these measures provide the greatest postural differences and were considered to be the most sensitive measure of orthostasis. Participants were also excluded if they reported use of any psychoactive drugs within the past week (e.g., marijuana, cocaine, ecstasy, psilocybin, phencyclidine, and ketamine), any alcohol consumed within the previous 12 h, and any caffeine or nicotine consumed within the previous 3 h for each float. For all other medications, participants were required to be stably medicated prior to participation, defined as having taken the medication for 6 weeks or longer. Participants were also screened for a history of unstable liver or renal insufficiency, glaucoma, diabetes, significant and unstable cardiac, vascular, pulmonary, gastrointestinal, endocrine, neurologic, hematologic, dermatologic, rheumatologic, or metabolic disturbance and excluded if they reported any of these conditions. All study procedures were approved by the Western Institutional Review Board. All participants provided written informed consent prior to participation and received compensation for study participation.

### Clinical Assessments

After providing informed consent, all participants underwent diagnostic verification *via* a clinical history and evaluation by a board certified psychiatrist (SSK or SEM) with the application of DSM-5 criteria (American Psychiatric Association, 2013). During this session, participants completed a measurement of their BMI, medical history, medication assessment, and vital sign measurements including orthostatic BP.

### Experimental Protocol

In this single group open-label pre–post-study design, all participants were provided access to four sequential REST sessions involving supine floating: reclining in a comfortable zero-gravity chair (chair-REST), followed on three occasions by floating in a pool of water (floatation-REST). The pool floats also followed a sequential protocol whereby participants first floated in an open pool (floatation-*REST*, open) before floating in an identically sized pool with an enclosure (floatation-*REST*, enclosed). For each session, participants were encouraged to float for the full 90 min, but they could also stop the experience at any time. These procedures were intended to help participants accommodate to the float environment and to ensure they were in full control over the experience. Sessions were spaced approximately 1 week apart. To ensure there were no external distractions, participants removed all personal belongings (including cellular phones) before each float.

#### Session 1: Chair-REST

**FIGURE 1:**
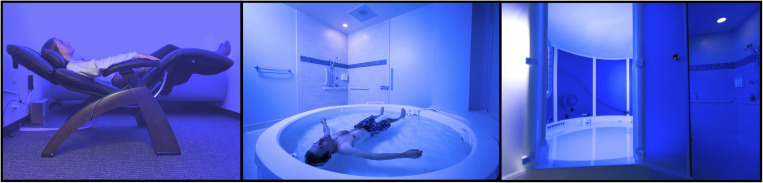
REST at LIBR. **(Left)** Chair-REST. **(Middle)** Floatation-REST in an open pool. **(Right)** Floatation-REST in an enclosed pool. The open **(middle)** and enclosed **(right)** pools are both 2.44 m in diameter and 0.28 m in depth. The domed pool contains an enclosure with a 2.44-m domed ceiling. Each pool contains 11 in. of reverse osmosis water saturated with ∼816 kg of USP grade Epsom salt (magnesium sulfate), creating a dense saltwater solution that is maintained at a specific gravity of ∼1.26, allowing participants to effortlessly float on their back while the water hovers just above the ears. The temperature of the water and the temperature of the air are both calibrated to match the temperature of the skin (∼35.0°C). For more details about how our float pools were engineered to minimize exteroceptive sensory stimulation to the nervous system, please see Feinstein et al. (2018a; 2018b).

Participants first reclined in a zero-gravity chair (Human Touch Perfect Chair PC510, Classic Power, Series 2) in the supine position for up to 90 min (Figure 1). The chair was ergonomically designed to take pressure off the spinal cord and contained memory foam backing to help the chair conform to each participant’s body shape. A motorized lever allowed the participant to recline the chair to a comfortable position. The chair was located in a dimly lit room using the same light as that used in the float pool. Participants could turn the light on and off using an infrared air switch. Participants remained clothed throughout (unlike the typical floatation-REST procedure, in which individuals are typically naked), and consequently, the room was maintained at a normal room temperature of approximately 23.3°C.

#### Session 2: Floatation-REST, Open

Participants floated in a supine position for up to 90 min in an open circular fiberglass pool (2.44 m diameter, 0.28 m depth), custom designed for research purposes by Floataway (Norfolk, United Kingdom) (Figure 1), a design that we selected to optimize the float experience for individuals with heightened anxiety and claustrophobia (see Feinstein et al., 2018b). The pool contained water filled with approximately 816 kg of USP-grade Epsom salt (magnesium sulfate), creating a dense saltwater solution maintained at a specific gravity of ∼1.26, allowing participants to effortlessly float on their back. The room around the pool was constructed to be waterproof, soundproof, lightproof, and temperature controlled. Silent heaters were placed under the pool to maintain the water at a constant temperature and a dedicated heating, ventilation, and air conditioning system maintained the air at a constant temperature. The temperature of the water and air approximated the surface temperature of the skin (∼35.0°C), and could be adjusted remotely by the experimenter in a nearby control room. An intercom system allowed the participant to freely communicate with the experimenter throughout the float session should any issues arise, and specialized speakers placed around the perimeter of the pool allowed the experimenter to communicate with the participant and play music to signal the end of the session.

#### Sessions 3 and 4: Floatation-REST, Enclosed

Participants then floated in a supine position for up to 90 min in an enclosed pool with the same dimensions as the open pool (2.44 m diameter, 0.28 m depth) that was also fitted with a rounded wall and a 2.44-m domed ceiling. We included the enclosed condition as this procedure reflects the manner in which most recreational float pools are designed. The experience between the open and enclosed pools was essentially identical, with the exception that the enclosed pool allowed us to calibrate humidity with greater precision. Thus, the room dimensions, temperature controls, and intercom system for this room were identical to the open pool (see Figure 1 for a visual comparison of both pools). No physiological recordings were made during session 3 to allow each participant one “naturalistic” experience of the floatation-REST environment without any concurrent physiological measurements.

### Outcome Measures

#### Blood Pressure Measurements

During each session, orthostatic BP was measured before and after each float. Prior to initiating the first BP measurement, participants were asked to lay quietly for 5 min, to ensure that they had accommodated to their true resting physiological state for that session. Following the first lying BP measurement, all other measurements were taken after a 1-min delay during each position (sitting, then standing) to ensure that participants had equilibrated to the position, and so that readings were not artificially influenced by physical activity. Prefloat BP measurements were taken with a clinical-standard device CASMED 740 (CAS Medical Systems Inc., Branford, CT, United States) that is commonly utilized for vital sign measurement in inpatient hospital settings. In order to obtain BP measurements during the float environment, we used the QardioArm wireless BP monitor (Qardio Inc., San Francisco, CA, United States), an FDA-cleared automated sphygmomanometer which uses the Oscillometric method to achieve a measurement range of 40–250 mmHg and an accuracy of ±3 mmHg. The QardioArm has been clinically validated according to ANSI/AAMI/ISO 81060–2:2009 as well as the European Society for Hypertension International Protocol Revision 2010 (O’Brien et al., 2010). Thus, each participant underwent orthostatic BP measurement with both the CASMED and QardioArm devices during the prefloat condition, to ensure comparability between the devices. For each measurement, the BP cuff was positioned approximately 1 in. above the elbow, so that it was situated at the same level as the heart. During each float, participants were instructed to keep their arms positioned along the side of their body. A LimbO Waterproof Protector (Limbo USA, Portland, ME, United States) was placed over the QardioArm BP device in order to prevent water from reaching it during the pool float sessions (in which case all QardioArm BP measures were obtained with this sleeve applied, to avoid any external influences on the pre- and post-float data points). After collecting orthostatic BP measurements with the QardioArm, to characterize BP responses during the float, nine additional BP measurements were collected once every 10 min (less if the participant exited the float before the full 90 min)^1^. The initial BP measurement of this sequence occurred approximately 5 min into the float session. All BP data from the QardioArm was wirelessly transmitted in real time *via* Bluetooth 4.0 to an iPad tablet located in the adjacent control room. Each BP measurement took 30–60 s to complete and was initiated remotely by the experimenter using an application on the iPad. Since application of an intermittently inflating BP cuff represents a departure from the naturalistic experience of floatation-REST, after completing their first pool float participants were given the opportunity to experience the next float without any instrumentation applied. As a result, pre- and post-float BP data were available for only three sessions: the chair float, the first pool float, and the third (final) pool float.

#### Self-Report Measures

**FIGURE 2:**
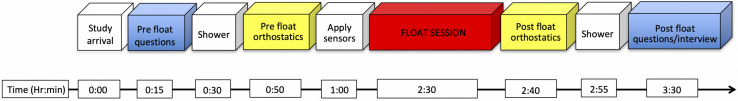
Approximate timeline of assessments during each REST session. Sensor application included the application of a wireless blood pressure monitor and waterproof cast. The total duration of each session was approximately 3.5 h.

All self-report measurements were administered electronically to participants *via* an electronic tablet (Apple iPad). Survey measures were obtained using Research Electronic Data Capture (REDCap^2^), a secure Web-based application for electronic collection and management of research and clinical trial data. All self-report measures were administered during the pre- and post-REST time periods, before and after the primary outcome measures had been collected (Figure 2), for each of the four sessions. Several different types of self-report measures were administered before and after each session of REST, as described next.

#### State-Trait Anxiety Inventory-State Form

The Spielberger State Anxiety Inventory (Spielberger et al., 1983) is a widely used 20-item self-report questionnaire intended to assess an individual’s level of anxiety at the present moment with total scores ranging from 20 to 80. The items assess for the presence or absence of current anxiety symptoms, and the measure has been shown to have excellent internal consistency and good convergent and discriminant validity (Spielberger et al., 1983). Participants completed the State-Trait Anxiety Inventory (STAI) immediately before and after each REST session.

#### Positive and Negative Affect Schedule*—*Expanded Form

The Positive and Negative Affect Schedule—Expanded Form (PANAS-X) (Watson et al., 1988) is one of the most commonly used measures of mood, with high internal consistency, and good convergent, discriminant, and construct validity. We chose the expanded form, which has several subscales measuring general dimensions of affect (positive and negative), as well as basic emotions (e.g., fear, sadness, joviality, etc.). We included the negative affect, positive affect, joviality, fatigue, and serenity subscales. Each subscale uses the same 5-point Likert-type response scale to collect ratings, ranging from 1 (very slightly or not at all) to 5 (extremely). The positive affect subscale has participants rate how *active*, *alert*, *attentive*, *determined*, *enthusiastic*, *excited*, *inspired*, *interested*, *proud*, *and strong* they feel at the present moment. The negative affect subscale has participants rate how *afraid*, *scared*, *nervous, jittery*, *irritable*, *hostile*, *guilty*, *ashamed*, *upset*, *and distressed* they feel at the present moment. The joviality subscale has participants rate how *happy*, *joyful*, *delighted*, *cheerful*, *lively*, *and energetic* they feel at the present moment. The fatigue affect subscale has participants rate how *sleepy*, *tired*, *sluggish*, *and drowsy* they feel at the present moment. Finally, the serenity subscale has participants rate how *calm*, *relaxed*, and *at ease* they feel at the present moment. Participants completed the PANAS-X immediately before and after each REST session.

#### Visual Analog Scales

Participants completed several Visual Analog Scale (VAS) measures where they rated how they currently felt on a 100-point scale that went from 0 (Not at all/None) to 100 (Extremely/The most I have ever felt). Each scale contained a digital slider that participants could move along a horizontal axis. We included affective VAS measures for *relaxation*, *stress*, *refreshed*, and *energy*. The Relaxation VAS asked, “How relaxed do you feel right now?” The Stress VAS asked, “How stressed or anxious do you feel right now?” The Refreshed VAS asked, “How refreshed do you feel right now?” The Energy VAS asked, “How much energy do you have right now?” Participants completed the VAS measures immediately before and after each REST session.

#### Interoceptive Awareness Measures

Participants completed several interoceptive awareness VAS measures assessing the intensity of *heartbeat*, *breath*, *and stomach/digestive* sensations immediately before and after each REST session, on a 100-point scale that went from 0 (Not at all/None) to 100 (Extremely/The most I have ever felt). For the pre session questions, the heartbeat VAS asked, “How intensely do you feel your heartbeat right now?” The breath VAS asked, “How intensely do you feel your breath right now?” The stomach VAS asked, “How intensely do you feel your stomach or digestive system right now?” For the post session questions, the heartbeat VAS asked, “How intensely did you feel your heartbeat while floating?” The breath VAS asked, “How intensely did you feel your breath while floating?” The stomach VAS asked, “How intensely did you feel your stomach or digestive system while floating?”

#### Body Appreciation Scale 2

The Body Appreciation Scale (Tylka and Wood-Barcalow, 2015) was developed to assess an individual’s trait level of positive acceptance of attitudinal characteristics toward their body. The Body Appreciation Scale 2 (BAS-2) was modified from its original version (to remove sex-specific terms and body dissatisfaction-based language), with 10 items using a 5-point Likert scale from 1 (Never) to 5 (Always), with total scores ranging from 10 to 50. Participants completed the BAS-2 once prior to floating (at baseline), and again immediately following each REST session. As a trait measure, the BAS-2 trait instructs participants to “Please indicate whether the question is true about you never, seldom, sometimes, often or always.” However, since each REST session reflected a state measurement, we modified the post-REST instructions for the BAS-2 accordingly. Thus, the postsession measures instructed participants: “For each of the questions below, please rate how you felt during the float.”

#### Body Image States Scale

The BISS (Cash et al., 2002) was developed to assess an individual’s level of negative attitudinal characteristics toward their body, *via* six items that query feelings about physical appearance, body size, shape, weight, and attractiveness. Participants choose among nine options that best describe how they feel “right now at this very moment” from “Extremely dissatisfied” to “Extremely satisfied” (or *vice versa*), with total scores computed as the mean of the six items after reverse scoring three of the positive-to-negative items. Participants completed the BISS immediately before and after each REST session.

#### Photographic Figure Rating Scale

The PFRS (Swami et al., 2008) was developed to assess an individual’s visual perceptual preference for different body types. It consists of 10 photographic images of different women (with the head obscured), each with BMI measurements that vary from emaciated to obese. Participants are instructed to select the body type that they perceive most accurately reflects (1) their current body type and (2) their ideal body type. A body dissatisfaction score is then calculated by subtracting the participant’s ideal self-rating from their current self-rating. The measure has been reported to demonstrate good construct validity (Swami et al., 2008) and good test–retest reliability on repeated administrations (Swami et al., 2012). Participants completed the PFRS immediately before and after each REST session.

#### Eating Disorder Examination Questionnaire 6.0

The Eating Disorder Examination Questionnaire 6.0 (EDE-Q 6.0) (Fairburn and Beglin, 2008) is a commonly used 28-item self-report measure based on the eating disorder examination interview used by clinicians to diagnose and assess severity of an eating disorder. The EDE-Q 6.0 consists of a global eating disorder score (total of four facets averaged), as well as four groups of eating disorder symptoms, which include eating restraint, eating concerns, shape concerns, and weight concerns, with each ranging from a score of 0 to 6 (higher scores are considered to index greater illness severity, with normative studies suggesting that a score of 2.2 or greater is indicative of symptoms above the 65th percentile; Luce et al., 2008). For the purposes of this study, we collected and report EDE-Q 6.0 total scores during the initial prefloat baseline assessment in order to provide an indication of self-reported illness severity.

#### Presession Instructions

Prior to each session, all participants were instructed that they could remain in the session “for up to 90 min” and that they could stop at any time. During each session, participants were encouraged to try to remain still and to try not to fall asleep. Participants were encouraged to experience each session with the lights turned off, but they were also reminded that they were free to turn the lights on whenever they needed them. The full instruction set can be found in the Supplementary Material.

#### Postsession Interviews

After each REST session, participants completed a debriefing interview with an experimenter to assess their experience. Questions were open-ended and asked about how the session went, their general experience, as well as questions about positive and negative thoughts or experiences during each session. These interviews were recorded and transcribed.

### Setting

All assessments were conducted at the Laureate Institute for Brain Research facilities.

### Sample Size Calculation

Sample size was estimated based on the primary outcome (orthostatic hypotension). We intended to obtain an estimate on the proportion of orthostatic hypotension with a margin of error <15%. Assuming no participant would show orthostatic hypotension, we calculated that *N* = 19 participants would provide an exact one-sided 95% confidence interval of 14.6%. Our recruitment assumed a 20% incompletion rate (i.e., 23 participants needed).

### Statistical Analysis

#### Primary Outcome

The standing-vs.-lying changes in BP were calculated for each participant, and the proportion of participants demonstrating orthostatic hypotension was evaluated using the exact binomial method.

#### Secondary Outcomes

The secondary outcomes were obtained on the same participants repeatedly across REST sessions, which were analyzed using LMM. Three different LMM’s were built according to the availability of outcome measures, with the inference of interest focused on time of floating (for BP) or post-vs.-pre-REST changes (for all other secondary outcomes). Before assessing the secondary outcome measure of BP, we evaluated the reliability of the QardioArm vs. CASMED devices was evaluated for each session and each position using the Pearson correlation coefficient, and for all sessions and positions combined using the intraclass coefficient (ICC), determined by the sum of between-subject random-effect variance components divided by the total (between- and within-subject) random variance components obtained from a random-effects model with fixed intercept and random intercepts of subject, session, and measure (lying, sitting, and standing). The secondary outcome of BP measures were obtained during REST and were modeled by a LMM with session, time, and/or session-by-time interaction as potential fixed effects, and random subject and/or session intercepts, with a potential first-order autoregressive (AR1) correlation structure. For each of the SBP and DBP measures, we fitted three fixed-effects options (time only; time and Session main effects; main effects plus time and Session interaction) × 5 random-effects/correlation-structure options (random subject intercepts only; random session intercepts only; random subject and session intercepts; random subject and session intercepts plus random time slope; random subject and session intercepts plus AR1 correlation structure) = 15 combinations. The final models of optimal fixed and random effects were chosen by the smallest values of Bayesian Information Criterion (BIC). To make all other secondary outcome measures consistent in scale, each participant’s raw and change scores for state anxiety, affect, body image disturbance, and interoceptive awareness were first converted into standardized units representing the percent of maximum possible (POMP) for each measure ranging from 0 to 100% (Cohen et al., 1999), a procedure modeled after our previous study (Feinstein et al., 2018a). This step made all measures in the same scale as VAS ranging from 0 to 100%. For all secondary outcome measures (except the BAS-2 scale), these POMP scores were then fitted to a 2nd LMM with fixed session (pre vs. post) effect and random subject and session intercepts. The inference of interests was on the post-vs.-pre-POMP changes.

For the BAS-2 scale, its POMP was fitted to a 3rd LMM with 2 fixed-effect options [intercept only; session (baseline, Chair-REST, Flotation-pool open, Flotation-pool enclosed 1 and 2) main effect] × 3 random-effects/correlation-structure options (random subject intercept; random session intercept, random subject intercept and AR1 correlation structure across sessions) = 6 combinations. As before, the optimal fixed- and random-effects/correlation structure was determined by BIC.

Parameters in all LMMs were obtained using the restricted maximum likelihood (ReML) method, and the degrees of freedom were calculated using the Kenward–Roger method for LMM with only random effects but no AR1 correlation. The 95% confidence intervals (CIs) of the fixed-effect parameters were calculated by either the approximate method assuming normal distributions for the fixed-effects when AR1 correlation structure was evaluated, or otherwise by the percentile method using 500 parametric bootstrap samples. The overall time or session effects (i.e., variables with more than two levels) were assessed by the *F*-test *via* type III analysis of variance. Considering the early phase of the study and exploratory nature of the measures, no procedure was applied for multiple comparisons, and our inference focused on point and interval estimates. The whole analysis was performed on R version 3.5.2, using R packages lme4 version 1.1-21 (Bates et al., 2015) and nlme version 3.1-137 (Pinheiro et al., 2018) for LMM, lmerTest version 3.1-0 for the Kenward–Roger method (Kuznetsova et al., 2017), and emmeans version 1.3.5 for marginal means (Lenth, 2019).

## Results

### Participant Demographics

Demographic characteristics for the 23 recruited partially weight-restored AN participants are listed in Table 1. During the structured clinical interview, the lifetime history of AN as defined by DSM-5 criteria was confirmed for all participants. The group showed an average age of onset of 15 years, an average illness duration of 9 years, and an average lowest self-reported BMI of 15.2. Although the current BMI average for the group was in the normal range, there was evidence of residual AN symptoms based on higher-than-normal EDE-Q global scores and elevated trait anxiety on the STAI-trait scale (Table 1).

**TABLE 1:** Demographics for the AN participant group.

**Demographic**	**AN participants**
Age	26.6 ± 9 years
Sex	22 females, 1 male
Education	14.9 ± 2.6 years
Age of illness onset	15.9 ± 4.9 years
Illness duration	9.0 ± 6.1 years
Lowest BMI	15.2 ± 1.9 units
Current Body mass index (BMI)	21.8 ± 2.7 units
Eating Disorder Examination Questionnaire Total score (range, 0–6)	2.26 ± 1.4 units
Spielberger Trait Anxiety Inventory (range, 20–80)	54.1 ± 8.8 units

*Means ± standard deviation. AN, anorexia nervosa.*

### Completion Rate

**FIGURE 3:**
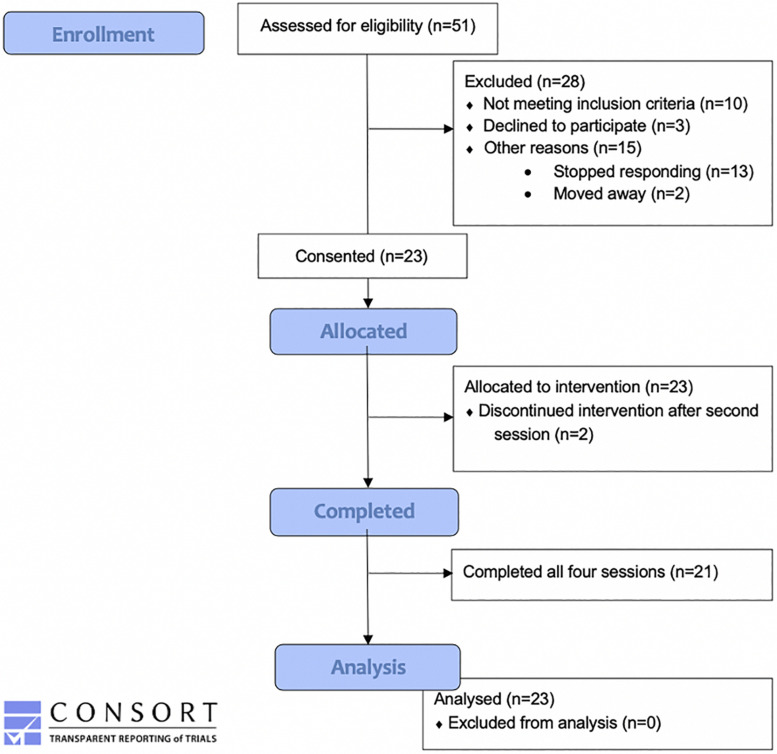
Consort flow diagram. This figure illustrates the number of participants included in enrollment, the number allocated to the REST intervention, the number withdrawn, and the number included in the data analysis.

All 23 of the participants completed the chair and the open-pool sessions, but only 21 of them completed the two enclosed-pool sessions (see Figure 3 for CONSORT diagram). Of the two participants who withdrew after the second session, one cited a lack of interest in the float pool environment, and the other did not give a reason and stopped responding to appointment requests.

### Session Duration

The median and IQR (in bracket parentheses) of each session duration was 90 [82, 90], 90 [85, 90], 90 [90, 91], and 90 [85, 90] min for chair, open pool, and the two enclosed pool sessions, respectively (see Supplementary Material), demonstrating that participants were able to tolerate the 90 min sessions.

### Reliability Check for Blood Pressure Measurement

Across all sessions and positions, the median and IQR of the Pearson correlation coefficients measured between the QardioArm and CASMED devices was 0.80 [0.75, 0.83] and 0.80 [0.73, 0.83] for SBP and DBP, respectively (see Supplementary Material for details). When all sessions and positions were considered together, the ICC for systolic BP was 0.70 and for diastolic BP was 0.73, indicating good reliability across devices.

### Primary Outcome of Orthostatic Hypotension

**FIGURE 4:**
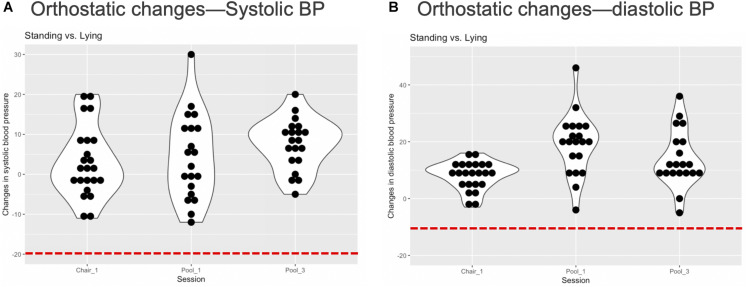
Individual orthostatic blood pressure changes. **(A)** The figure displays each individual’s change in systolic blood pressure (BP) from lying to standing at the end of each REST session. There was no evidence of systolic orthostatic hypotension in any individual, i.e., a reduction in systolic BP below 20 mmHg (red dashed line). **(B)** The figure displays each individual’s change in diastolic BP from lying to standing at the end of each REST session. There was no evidence of diastolic orthostatic hypotension in any individual, i.e., a reduction in systolic BP below 10 mmHg (red dashed line). Note that BP was not measured during the second pool session and is therefore unlisted.

**FIGURE 5:**
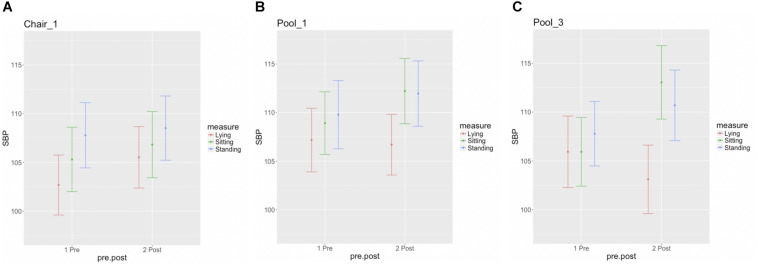
Group summary of orthostatic blood pressure measures. Each figure displays the mean and standard error for the lying, sitting, and standing blood pressure measurements, at pre- and postsession intervals for the **(A)** chair-REST **(B)** the first and **(C)** final floatation-REST sessions.

None of the participants completing any float session exhibited evidence of meeting the established criteria for orthostatic hypotension, suggesting a one-sided 95% CI of 13% (upper tail) in this population. Figure 4 displays the postfloat orthostatic BP measurements for each individual. At the group level, the median and range (in bracket parentheses) of the systolic and diastolic BP changes from lying to standing was 5 [−12, 30] and 11 [−5, 46] mmHg, respectively, across the three sessions (Figure 5). With respect to other safety measures, we did not observe any falls upon standing, and there were no reports of feeling lightheaded or dizzy. Additionally, there were no adverse events such as acute panic attacks, severe dysphoria, agitation, or increased suicidal ideation.

### Secondary Outcomes

#### Blood Pressure Responses During REST

**FIGURE 6:**
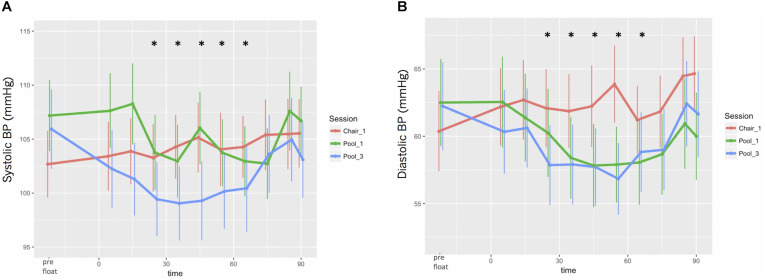
Blood pressure responses during REST. **(A)** Systolic BP. **(B)** Diastolic BP. Significant reductions in both systolic and diastolic BP occurred between the 25th and 65th minutes of REST.

Systolic BP appeared to slightly increase during Chair-REST but showed a U-shaped pattern (decreased, remained stable, and then increased) during floatation-REST, with the largest decrease occurring during the final session (Figure 6A). Nonetheless, the differences across sessions were not substantial, and BIC suggested a LMM with only the time but not the session nor the session-by-time interaction fixed-effects. The LMM suggested the largest reductions (with 95% CI, *t*-statistic, and *p* value) ranged between 2.3 [(0.47, 4.13), *t*(592) = −2.47, *p* = 0.014] and 3.7 [(1.83, 5.49), *t*(592) = −3.93, *p* < 0.0001] mmHg between the 25th and 65th minutes of REST. Diastolic BP showed a more consistent decrease across both sessions of floatation-REST as compared with chair-REST (Figure 6B) and BIC also selected a LMM with only time as a fixed-effect. The LMM suggested the largest reductions ranged between 1.8 [(0.17, 3.48), *t*(592) = −2.17, *p* = 0.030] and 2.6 [(0.83, 4.39), *t*(592) = −2.88, *p* = 0.004] mmHg between the 25th and 65th minutes of REST.

#### State-Trait Anxiety Inventory-State Form

For the secondary outcome of STAI-state anxiety, the LMM suggested that the post-vs.-pre-POMP change was on average −15.2% (with a 95% CI of −18.3 to −12.1%) across all four REST sessions (*p* < 0.0001).

#### Positive and Negative Affect Schedule—Expanded Form

For the secondary outcome of PANAS negative affect, the LMM suggested that the post-vs.-pre-POMP change was on average −8.3% (with a 95% CI of −11.2 to −5.3%) across all four sessions (*p* < 0.0001). For the secondary outcome of PANAS positive affect, the LMM suggested that the post-vs.-pre-POMP change was on average 4.5% (with a 95% CI of 1.2 to 7.9%) across all four sessions (*p* = 0.007). For the secondary outcome of PANAS joviality, the LMM suggested that the post-vs.-pre-POMP change was on average 5.5% (with a 95% CI of 1.9–9.8%) across all four sessions (*p* = 0.007). For the secondary outcome of PANAS fatigue, the LMM suggested that the post-vs.-pre-POMP change was on average −13.9% (with a 95% CI of −20.2 to −8.4%) across all four sessions (*p* < 0.0001). For the secondary outcome of PANAS serenity, the LMM suggested that the post-vs.-pre-POMP change was on average 25.3% (with a 95% CI of 19.8, 31.5%) across all four sessions (*p* < 0.0001). Thus, participants reported reduced negative affect and fatigue from pre- to post-REST, whereas they reported increased positive affect, joviality, and serenity from pre- to post-REST.

#### Visual Analog Scales

For the secondary outcome of VAS relaxation, the LMM suggested that the post*-*vs.-pre-POMP change was on average 21.9% (with a 95% CI of 17.3–26.1%) across all four sessions (*p* < 0.0001). For the secondary outcome of VAS stress, the LMM suggested that the post-vs.-pre-POMP change was on average −22.9% (with a 95% CI of −27.3 to −18.5%) across all four sessions (*p* < 0.0001). For the secondary outcome of VAS refreshed, the LMM suggested that the post-vs.-pre-POMP change was on average 32.0% (with a 95% CI of 27.0–36.4%) across all four sessions (*p* < 0.0001). For the secondary outcome of VAS energy, the LMM suggested that the post-vs.-pre-POMP change was on average 16.6% (with a 95% CI of 12.0–20.6%) across all four sessions (*p* < 0.0001). Thus participants reported reduced stress from pre- to post-REST, whereas they reported increased relaxation, refreshment, and energy from pre- to post-REST.

#### Interoceptive Awareness Measures

For the secondary outcome of heartbeat intensity, the LMM suggested that the REST-vs.-baseline POMP change was on average 10.6% (with a 95% CI of 2.7–17.9%) across all four sessions (*p* = 0.0032). For the secondary outcome of breath intensity, the LMM suggested that the REST-vs.-baseline POMP change was on average 8.8% (with a 95% CI of 2.7–14.8%) across all four sessions (*p* = 0.0043). For the secondary outcome of stomach/digestive intensity, the LMM suggested that the REST-vs.-baseline POMP change was on average −1.8% (with a 95% CI of −8.7–5.1%) across all four sessions (*p* = 0.581). Thus, during REST, participants reported feeling significant increases in the intensity of the sensations from their heartbeat and breath but not from their stomach/digestive system.

#### Body Appreciation Scale 2

For the BAS-2, the score increased by 0.26 (95% CI from −0.06 to 0.58), 0.10 (from −0.28 to 0.48), 0.10 (from −0.32 to 0.51), and 0.08 (from −0.34 to 0.50) across the four sessions of REST. The type III analysis of variance comparing the post-float assessments vs. the initial baseline assessment did not observe a statistically significant difference across sessions: *F*(4,84) = 1.38, *p* = 0.25, and BIC suggested a model without a session effect.

#### Body Image States Scale

For the BISS, a negative attitudinal body image secondary outcome measure, the LMM suggested that the post-vs.-pre-POMP change was on average 1.05% (with a 95% CI of 0.7–1.5%) across all four REST sessions (*p* < 0.0001), suggesting a statistically significant increase in more favorable body image state.

#### Photographic Figure Rating Scale

For body dissatisfaction ratings on the PFRS, a visual perceptual body image secondary outcome measure, the LMM suggested that the post-vs.-pre-POMP change was on average −4.66% (with a 95% CI of −6.58 to −2.67%) across all four float sessions (*p* < 0.0001). Thus, participants reported significantly reduced body image dissatisfaction from pre- to post-REST.

#### Effect Sizes for Secondary and Exploratory Outcome Measures

**FIGURE 7:**
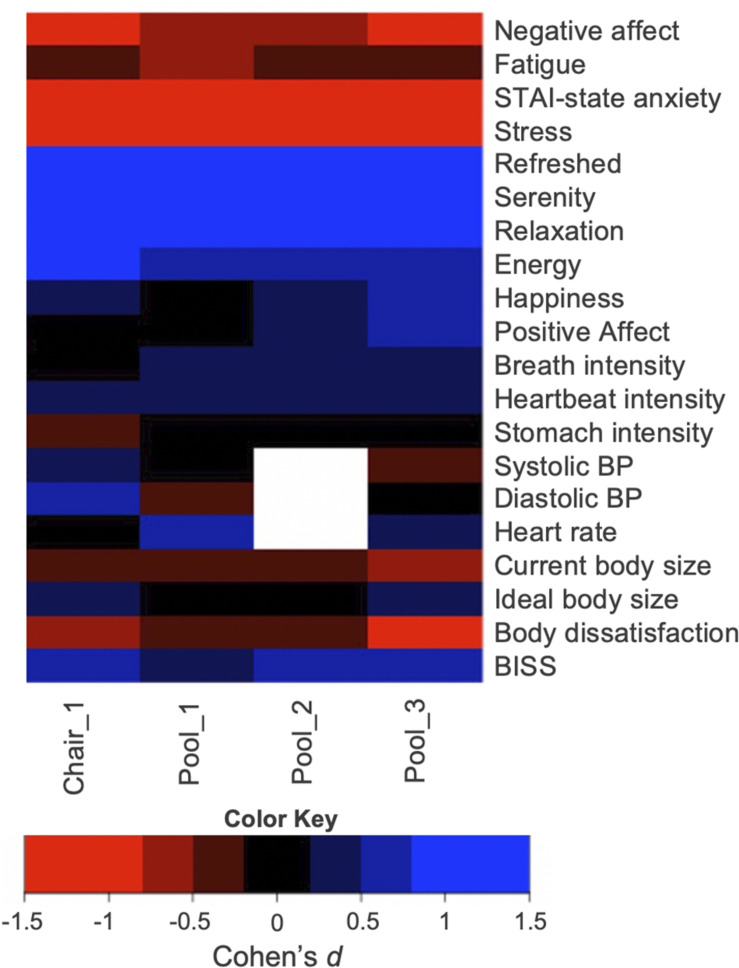
Effect size estimates for the secondary outcome measures. Note that there were no blood pressure measures taken during the second pool float, and therefore that field is blank. A table showing the exact effect size estimates and associated confidence intervals is located in the Supplementary Material.

Effect size calculations (Cohen’s *d*) for all secondary and exploratory outcome measures are listed in Figure 7. REST elicited moderate (0.5) to large (0.8 and greater) effects on ratings of state anxiety, stress, refreshment, serenity, relaxation, energy, and PFRS body dissatisfaction. REST elicited small (0.2) to moderate (0.5) effects on BP, heartbeat and breath intensity, and negative attitudinal body image. REST appeared to have minimal effects on stomach/gastrointestinal sensation intensity ratings and positive body appreciation ratings.

#### Postsession Interviews

A complete transcription of the post-REST interviews is provided in the Supplementary Material. Overall, most participants found the REST experience to be positive. Many participants stated that the idea of the REST environment elicited some apprehension, particularly during the initial minutes of exposure to REST or when they turned the lights off (in which case they kept the lights on during the session). These feelings were usually followed by a sense of physical relaxation and a slowing of their thought process. Many individuals spontaneously reported experiencing their heartbeat and breathing sensations more intensely, as well as a sense of weightlessness, particularly in the floatation pools. Many found that the REST environment elicited thoughts about their bodies and body image concerns, with a couple of individuals reporting performing body checking maneuvers, but more individuals noted that they experienced a positive change in their relation to their bodies during REST. For example, some individuals reported positively experiencing a sense that they could not feel their outer body limits or their stomach sensations at certain times, eventually moving their limbs or trunk to re-engage the sense of body ownership. A few reported consciously noting that their sense of distress and focus about their body image was lower during the sessions. When directly queried, there were more positive endorsements of the REST environments during each float than there were negative endorsements.

## Discussion

We conducted this single group open-label study with the primary aim of investigating the safety and tolerability of REST in AN. Secondarily, we aimed to explore the impact of REST on affective symptoms, body image disturbance, and interoception. Prior to the current study, there have been no studies documenting the safety or tolerability of the procedure in any eating disorders population, and we could find only one brief theoretical review of the topic (Barabasz, 1993). Therefore, this study represents the first empirical investigation of REST in eating disorders.

With respect to the primary outcome of safety, we did not observe any evidence of orthostatic BP reductions for either the systolic or diastolic measure. We also did not observe any falls or other signs of adverse experiences such as acute panic attacks, severe dysphoria, agitation, or suicidal ideation in response to the REST environment. Some participants reported mild apprehension with exposure to the dark, with thoughts about their body image, or with worry about having increased anxiety in the REST environment, but ultimately these were felt to be tolerable as evidenced by the significant reduction in state anxiety, comments provided during postsession interviews, and high rate of study completion (21 out of 23 individuals completing the entire four session protocol). Collectively, these results suggest that the participants in this study safely tolerated the REST environment and found it to be acceptable. The sample recruited for this study was composed of outpatients with a lifetime diagnosis of AN, with moderate levels of residual symptoms according to their scores on the EDE-Q, and heightened levels of trait anxiety according to the STAI. To reduce the risk of falling while in an unsupervised environment, future studies of more acutely ill individuals (e.g., inpatients) would need to demonstrate that they do not exhibit signs of orthostatic hypotension before commencing REST.

With respect to the secondary outcome measures, we observed significant reductions in several affective measures from pre- to post-float including self-reported state anxiety, stress, fatigue, and negative affect, as well as significantly increased positive affect, relaxation, joviality, refreshment, energy, and serenity. The observed reductions in state anxiety on the STAI are potentially noteworthy for several reasons: (1) current anxiolytic medications such as benzodiazepines (Steinglass et al., 2014) and behavioral treatments for anxiety in AN show limited efficacy, (2) the magnitude of the effect was large, and (3) they mirror our previous observation of acute anxiety reductions in a transdiagnostic group of individuals with heightened anxiety sensitivity (Feinstein et al., 2018a). At this juncture, it is important to emphasize that such observations are to be regarded as preliminary until they can be verified in subsequent studies employing control conditions and randomized participant assignment, to account for the potential impact of expectancies on responses to this novel behavioral intervention.

In the current study, we observed significant reductions in body dissatisfaction measured *via* the PFRS, with effect sizes ranging from small during the first few floats to large during the final float. This amounted to a POMP reduction of nearly 5%. We also observed an improvement in the BISS, a negative attitudinal measure of body image disturbance but to a much smaller extent (only about 1% POMP). There was also no effect of floating on the BAS, a positive attitudinal measure of body image. These results are important because it is well known that individuals with AN often retain body image disturbances long after achieving weight restoration, and body image disturbance has been identified as a predictor of relapse (Keel et al., 2005). Explanatory models of AN have traditionally focused on the roles of personality traits [e.g., obsessionality (Lilenfeld et al., 2006), perfectionism (Zucker et al., 2011), cognitive inflexibility (Friederich and Herzog, 2011)], culturally derived values [e.g., “thinness ideal” (Levine and Smolak, 2010)], or family environment (Klump et al., 2002). While such models have revealed key aspects of eating disorders, it has been suggested that treatments directed toward them yield only moderate recovery rates (Pike, 1998; Couturier et al., 2013; Galsworthy-Francis and Allan, 2014; Tchanturia et al., 2014), indicating that research into novel therapies is needed. The possibility that REST could have a therapeutic impact on measures of body image is potentially noteworthy, but it should also be noted that the observed effects were short term and were not compared against a control intervention.

We also observed significant changes in interoceptive awareness for heartbeat and breathing sensations (with a medium effect size) but not for the stomach/digestive system. Increased cardiorespiratory sensation changes during floatation-REST mirrored our previous observation with anxious individuals (Feinstein et al., 2018a), but the lack of change in stomach/digestive system changes was noteworthy given the predominant focus on such symptoms outside of meal times, such as fullness, bloating, and constipation (Robinson, 1989; Halmi and Sunday, 1991; Sato and Fukudo, 2015) in AN. While we cannot know whether this pattern of interoceptive changes is the source of their positive affective responses to REST, it would seem that changes in gastrointestinal (GI) sensation were not a major contributing factor. The fact that GI sensations were actually diminished during REST (and not heightened like other interoceptive sensations) is also important from a safety perspective, as it highlights that REST does not seem to exacerbate the intensity of their already uncomfortable GI sensations. Interestingly, several recent studies exclusively using self-report measures have suggested that interoceptive awareness may be positively associated with positive body image in adolescents and adults (Todd et al., 2019a,b). The current study extends this link *via* physical exposure of AN individuals to the REST environment, finding both increases in cardiorespiratory interoceptive sensations and improved positive body image measures.

### Limitations

This study has several limitations that elicit considerations for future investigations. For this initial study, we recruited a relatively small sample that was partially weight restored, was composed of individuals with a lifetime history of AN (i.e., some did not have a current diagnosis of AN), and was thoroughly screened to exclude the presence of acute medical illness. We therefore cannot rule out the possibility that a larger or more clinically heterogeneous sample might have had a different outcome with respect to safety, tolerability, and subjective outcome. The lack of a control condition in our use of an open-label design means that the observed results could be susceptible to the effects of expectation. Although such effects can impact any type of clinical trial, they are especially important to consider when conducting non-pharmacological clinical trials. In the current study, we attempted to constrain such expectations by providing participants with only the minimum information necessary for them to consider the potential risks and time constraints involved in study participation. We also decided against randomizing to a control condition in this initial study based on (1) our primary interest in gathering safety and feasibility data and (2) ensuring in this initial study a gradual exposure to REST so as to minimize potential safety risks from a more rapid immersion directly into floatation-REST. Thus our approach followed the development principles of behavioral clinical trials optimization [e.g., the ORBIT model (Czajkowski et al., 2015)], in which the early-phase (akin to Phase I) relates to ‘defining and refining’ the intervention, leading to future proof-of-concept and pilot feasibility studies (akin to Phase II), and finally, late-phase efficacy trials (akin to Phase III). Now that there are some safety data available, we recommend that future studies investigating the efficacy of REST in modifying clinically relevant outcomes incorporate a randomized control condition (e.g., a wait-list control group) or a crossover design (Feinstein et al., 2018a). Other comparator options include employing a usual care group or attentional control group (Freedland et al., 2011); these latter approaches may be preferable on an inpatient unit, where usual care for AN is intensive and includes multiple forms of treatment such as pharmacotherapy and psychotherapy. Another consideration is the finding that the chair-REST condition exhibited effect sizes that were similar to floatation-REST for some of the secondary outcome measures, such as negative affect, anxiety, stress, relaxation, serenity, and refreshment, potentially raising questions about whether exposure to the pool environment would even be necessary to elicit some of these effects. However, the observed effects on body image (particularly, body dissatisfaction on the PFRS), interoception, BP, as well as positive affect could indicate some specificity to the float pool environment. Future trials employing a randomized controlled design can help disentangle whether specific types of REST (Suedfeld and Borrie, 1999) are more efficacious than others for treating AN. Finally, a third limitation relates to the lack of control for multiple comparisons when examining the numerous secondary objectives. With these considerations in mind, we regard these results as preliminary and hypothesis generating for future studies. We have chosen the emphasize the observed effect sizes (which also must be interpreted with caution given the aforementioned lack of a control group to manage potential influences of expectancy), in hopes that they are of use in the design of future studies.

## Conclusion

Overall, the findings from this initial trial suggest that individuals with partially weight-restored AN can safely tolerate the physical effects of REST. They may also experience improvements in anxiety and body image disturbance, but further studies involving randomized controlled designs would be required to confirm this finding.

## Data Availability Statement

The raw data supporting the conclusions of this article will be made available by the authors, without undue reservation, to any qualified researcher.

## Ethics Statement

This study involving human participants was reviewed and approved by Western Institutional Review Board. All participants provided their written informed consent to participate in this study. Written informed consent was also obtained from the individual(s) for the publication of any potentially identifiable images or data included in this article.

## Author Contributions

SK and JF conceived the research idea, with input into the experimental design from SM, BP, VU, and MP. VU, RP, EB, and SC collected, collated, and transcribed the data. H-WY, SK, and JF analyzed the data. SK, JF, and H-WY drafted and edited the manuscript. All authors have read and approved the final manuscript before submission.

## Conflict of Interest

The authors declare that the research was conducted in the absence of any commercial or financial relationships that could be construed as a potential conflict of interest.

## Footnotes

1 Participants were not informed of the temporal intervals between BP measurements, to prevent them from estimating the duration of each float. Instead, they were informed they might feel the BP device inflate “periodically, from time to time.” Furthermore, the exact inflation interval was jittered randomly up to 1 min by the experimenter.

2 www.project-redcap.org

## Abbreviations

AN: Anorexia nervosa
BAS-2: Body Appreciation Scale Version 2
BISS: Body Image States Scale
BMI: Body Mass Index
BP: blood pressure
DBP: diastolic blood pressure
DSM-5: Diagnostic and Statistical Manual Version 5
EDE-Q: Eating Disorder Examination Questionnaire
ICC: intraclass correlation coefficient
IQR: interquartile range
LIBR: Laureate Institute for Brain Research
LMM: linear mixed-effects models
PANAS-X: Positive and Negative Affect Schedule Version X
POMP: Percent of Maximum Possible
PRFS: Photographic Figure Rating Scale
REST: Reduced Environmental Stimulation Therapy
SBP: systolic blood pressure
STAI: Spielberger Trait Anxiety Inventory.
