# Supplementary material for: Reduced Environmental Stimulation in Anorexia Nervosa: An Early-Phase Clinical Trial
Source: Front Psychol. 2020 Oct 6;11:567499. doi: 10.3389/fpsyg.2020.567499 (PMC7573249; doi:10.3389/fpsyg.2020.567499)
Supplement: Supplementary file 1 [file Data_Sheet_1.docx]

**Chair-REST (Session #1)^[[1]](#footnote-1)^**

**Q- In general, how did the float go?**

It was pretty pleasant. It was what I expected it to be. I mean it was about, it was no more pleasant than I expected or any less pleasant than just sitting in the chair and having the lights off.

-*PARTICIPANT 1*

Um, it was good.

-*PARTICIPANT 2*

Um, I think it went very well. Um, it was really enjoyable and relaxing (laughs).

-*PARTICIPANT 3*

Um… I think I forgot that the first time was gonna be like, just in the chair and then I thought maybe a chair in a pool, so…It was, um, it was okay. But I didn’t, I got a little antsy.

-*PARTICIPANT 4*

Pretty well. Do you want me to elaborate? [Yes please.] Okay. The beginning was really great. Then I ended up falling asleep for a little bit, and then I woke back up. And then it was still good. And then at the very end I was just like DONE. [How long do you think you felt done?] Probably 30 minutes, 20 minutes.

-*Participant 5*

I thought it was really fun. I really enjoyed it! (laughs)

-*PARTICIPANT 6*

Um, it went okay. At first I was really stressed out. And then I got so stressed out that I got tired. And then it felt like it lasted forever (laughs). [Why did you get stressed out?] I don’t know! I got like a lot of emotions all at once. Like when I first started. Like I got really panicked kind of. And sad. It was weird.

-*PARTICIPANT 7*

Great. It went really well. [Can you elaborate a little more?] Um, it was a lot more relaxing than I thought it was gonna be, just sitting in a chair. Lying in a chair. And, um, there were times where I did feel like, um, I was aware that I didn’t feel heavy. So, that was kind of a different experience.

-*PARTICIPANT 8*

Um, the float was comfortable and relaxing and I enjoyed the posture of laying back. My neck felt comfortable, um, but I did have to use the bathroom pretty significantly for probably three quarters of the float so I had to end early to go to the bathroom.

-*PARTICIPANT 9*

It went well! Um, it was comfortable, relaxing.

-*PARTICIPANT 10*

I think it went fine. Do you want more detail? (laughs) [Yes please!] Um, I slept, which I was trying not to, but I had to. I was worried about being bored or, I was worried about being stressed about other stuff I need to be doing and I didn’t feel that way so that was good.

-*PARTICIPANT 11*

It was okay. Not great. Kind of uncomfortable because of my back pain. And, just got distracted kind of easily and my mind was racing so I couldn’t really relax.

Q- What did you think about while floating that made your mind race?

A- I think just the boredom did, so…

-*PARTICIPANT 12*

It went really well! I felt very relaxed, to my surprise actually.

-*PARTICIPANT 13*

In general the float went pretty well. Um, felt pretty relaxed and at ease, especially in the beginning. Near the end I started to get kind of fidgety and a little anxious. Um, and just kind of like “okay I’m ready to go”. But overall it was pretty relaxing.

-*PARTICIPANT 14*

It went really well. Time went by faster than I expected it. It was pretty relaxing. I didn’t actually fall asleep fall asleep, but I was like super relaxed. I think I fell asleep anyway (laughs). But no I was really relaxed.

-*PARTICIPANT 15*

It was fine!

-*PARTICIPANT 16*

Good! Good, yeah.

-*PARTICIPANT 17*

Um…pretty well. I didn’t feel anything at first, but then I was relaxed and got to, um, enjoy my body I guess?

-*PARTICIPANT 18*

Um, pretty good. It was kind of different than I expected. Like I didn’t know once it started, if you were not present, like was I gonna feel something different? I thought there was like a button you were gonna push that was gonna make the chair feel like it disappeared? Um but that didn’t really happen but I did feel at some points like, I was kind of like floating, but not like…Like I could still feel the chair. So…it was kind of a weird, weird sensation.

-*PARTICIPANT 19*

It was good. I spent a lot of time worrying about who was supposed to be seeing anything at all, or not. Um…and the beginning, I don’t know when it stopped, I just felt like I was thinking a lot and I didn’t know if I was supposed to be thinking about something, make myself not think about anything, or what I should be thinking about. So that was like the very beginning and then somewhere in there it stopped or changed. [So you felt that you stopped worrying about it?] Mhmm. But I don’t know how long that was.

-*PARTICIPANT 20*

It went well.

-*PARTICIPANT 21*

Um, good. It was relaxing and comfortable. Um, it went well.

-*PARTICIPANT 22*

Think it went well. I don’t know how long I was in the chair but for the most part I felt really calm. [You were there for an hour.] That’s what I thought! That’s what I put on there.

-*PARTICIPANT 23*

**Open pool float (Session #2)^[[2]](#footnote-2)^**

**Q- In general, how did the float go?**

It was very pleasant, relaxing. Um… I did experience a desire like I said, to swim around because I knew I was in water, so I wanted to swim. But, it was relaxing just to lie there. By the end I was not as interested in being still and wanted to get out. But overall it was pleasant.

-*PARTICIPANT 1*

Um, it went really well. Yeah. Yeah it was (hard to relax) at the beginning and at the end, but the middle was good.

-*PARTICIPANT 2*

It was really good. It was a lot more enjoyable than the last one. Um, it was just really interesting. Um, I didn’t really know what to expect. I kind of lost my body, if that makes sense. So it was really neat.

-*PARTICIPANT 3*

It was pleasant. Um (laughs) I thought a lot about like my body position. So that was, it was interesting. I don’t know. I expected to not think about that as much.

-*PARTICIPANT 5*

Um, it was better than I thought it was gonna be.

-*PARTICIPANT 7*

Great! I really loved it (laughs). Feeling weightless.

-*PARTICIPANT 8*

Um, the float went well. It took a little while to get acclimated to the environment, being my first float ever. Um, but it was, it, after 20 or 30 minutes I definitely was calm and so I felt like, um, like it was an enjoyable experience.

-*PARTICIPANT 9*

It went really well.

-*PARTICIPANT 10*

It was wonderfully relaxing.

-*PARTICIPANT 12*

In general it went pretty well. I was pretty relaxed and, I don’t know, it was just a very unique experience. [What made it unique?] I don’t know. There’s numerous times where I couldn’t really feel my body. I couldn’t kind of feel where it started and then where the water was, and then I opened my eyes and I remember (mumbling) “oh and I can’t see anything either. And I can’t hear anything.” So I was like “okay, sensations!”

-*PARTICIPANT 14*

It went well.

-*PARTICIPANT 16*

Um, my experience was, um, pretty negative. Um I think when I had the lights off at first was when I was most panicked. Like too much going on in my head and not having any other senses around me, like not having my body. Um, like there was no safe place for me. So I did as much as I could with the lights off but then I needed to turn them on about halfway through or something… [And you just left them on when you turned them on?] Yeah. Yeah. And it was better after that. It wasn’t nearly as like—I mean I was crying for the whole time the lights were off. Um, so it was, it was scary for me.

-*PARTICIPANT 18*

Um it went by faster than the chair (laughs). And I, I enjoyed it. I didn’t really stay—the end part, like the last part, I stayed more still but in the beginning half I was kind of like, kind of like pushing myself back and forth I guess. So that was kind of fun, um, and yeah I liked it. The only thing that I didn’t like was, um, it took a while for my neck to get used to it. Had to like position itself so it wouldn’t be tight.

-*PARTICIPANT 19*

It was great! It was good.

-*PARTICIPANT 20*

It went well.

-*PARTICIPANT 21*

Um, I was pretty anxious. I was calm for part of the time but then—I was calm for a little while and then that went away and I got anxious. Yeah I would say like 20% of the time I felt good and that other 80% I was anxious.

-*PARTICIPANT 22*

It went really well. It was really relaxing and I was able to stay in for the whole time. And I would’ve stayed in longer too.

-*PARTICIPANT 23*

It was okay. It wasn’t as good as the first one… Um I think it was just more awkward I guess. I don’t know I just didn’t feel used to something like that. Well at first like keeping my head, like trying to like relax my neck and my head. Um, and then when I did I felt like the water was right, like at the center area of my face. But yeah I don’t know it just felt kinda long. It’s just something I’m not used to.

-*PARTICIPANT 17*

It went really well. I adjusted to it fairly…I don’t know it took about 20 minutes to…Ok so I was comfortable when I got in but then I felt nauseas for about 20 minutes after I laid back. Um and I was a little bit tense but then was able to relax pretty easily. The time went by faster than I thought it would. Um… it was extremely—I got out of the pool a lot more relaxed than when I got in. So overall it was really pleasant.

*-PARTICIPANT 15*

It went well. It was, it took a while to get relaxed, but I did feel like I eventually got to a very, very relaxed, pretty comfortable state.

-*PARTICIPANT 11*

Um, it was enjoyable in general. It was just kind of taking some getting used to. I felt like I was paralyzed for a few minutes. Kinda freaked me out. Yeah. Once I started to get into the floating, like I felt like, what if my body can’t move.

-*SUBJECT 4*

It went well! Took a little bit of getting used to at first, but…

-*PARTICIPANT 13*

(laughs) Not very well. It was not relaxing at all. I was anxious the entire time. So, eventually I just decided it wasn’t gonna get any better so I just got out before I panicked. Um, I really didn’t like not having any clothes on. Like that made me feel very vulnerable. And then I could just hear my heart, like pulsing in my ears and stuff, which made me just, I don’t know if that makes me more anxious because I feel like it was beating faster than it usually does. And then just like thinking about other people being in the pool without any clothes on and just like contamination stuff, and so I was like just ready to get out (laughs).

-*PARTICIPANT 6*

**Enclosed pool float (Session #3)^[[3]](#footnote-3)^**

**Q: In general, how did the float go?**

It was so miserable. I hated it so much. I wanted to get out almost the second I got in. Um… I’m trying to figure out why I would just, I didn’t want to be there. I felt anxious and when I tried to relax and um… you know, open up to the experience and so I mean I breathed and… um… did try to let myself go, but I didn’t, I didn’t enjoy it. And at one point… at one point kind of after I had been there probably half way through, I kind of started getting nerv—like, um…feeling claustrophobic. The air was heavy and I just… I mean I could breath, but it wasn’t… wasn’t pleasant.

-*PARTICIPANT 1*

Good.

-*PARTICIPANT 2*

It went really well. It was different than the other one. I couldn’t move a lot more. But I stayed awake the whole time. But yeah, I wouldn’t say it was better, but it was really positive.

-*PARTICIPANT 3*

It went better than the first time. I felt a little more comfortable. I was able to relax a little faster.

-*SUBJECT 4*

It went pretty well. Um, in the beginning I was really anxious but I was calm towards the end.

Q- What were you anxious about?

A- Um, I don’t know. I’m just not so good at sitting still so long. And it was closer to lunch time.

-*PARTICIPANT 5*

It was--it was good. I wasn’t anxious like I was last time. I was pretty calm. But not as calm as the chair.

-*PARTICIPANT 6*

It was great. And it went by really fast so I loved it.

-*PARTICIPANT 8*

The float went well. It was easier than the first tank float just because I kind of knew what to expect. And I had an overall positive experience.

-*PARTICIPANT 9*

It went well. I mean, about the same as last time. I thought—I think I was a little more antsy in the middle this time than last time.

-*PARTICIPANT 10*

It was relaxing and easier than last time to really get comfortable and feel just really relaxed. I didn’t have that moment of panic at the end. I, just whatever that panic, I did want to turn the light off and I did just for a second, but I didn’t want to keep it off because I was afraid of having that feeling at the end. And it wasn’t that it bothered me to have the light off. I liked that better and really wanted to keep it that way, but it was that feeling, or that concern, that when I was floating I would get separated from it and then, kind of just be in that almost disconnected place and want to turn it off and not, and then be disoriented and not be where I was or be able to get to it. So, I thought it would be really nice if I could just hold that thing I saw hanging there or a little button that I knew would be easy to turn it off and on. I probably would’ve kept it off. It was mostly with my eyes closed, so that was still good.

-*PARTICIPANT 11*

Initially, it was really relaxing and I liked the atmosphere, the construct, and the build in this particular float room. Um… but my towel fell in the water, got wet, the salt got in my eyes, I have terrible hangnails so I was constantly distracted and I had to be mindful of keeping my hands above the water instead of focusing on truly relaxing. So I was really distracted. I was able to hold on to some straps for a little while and float, you know, in the dark. But I did get, you know, water in my eye and it was just ample on my mind. And so in comparison to the last one it was not near as good, but, that’s more on me and my hangnails.

-*PARTICIPANT 12*

It went well!

-*PARTICIPANT 13*

In general it went okay. I was a little more anxious this time than the last float.

Q- Okay. Do you know why, or…?

A - I don’t know. I feel like at first the lights in the room were still on. And so, I don’t know I felt, like, I don’t know almost claustrophobic because I could, like, see the dome over me. I just felt like, “Okay, it’s kinda hard to breathe in here.” I was just a little more anxious, but once the lights went out I felt way better.

-*PARTICIPANT 14*

It went really well. The lights to the outside bathroom were on for part of it so it wasn’t completely dark but it was still really relaxing. I almost wonder if that was why I didn’t feel as like dizzy or nauseas at first because I wasn’t as disoriented? Kind of helped me transition in a little bit. And, I didn’t—so compared to the last float, I wasn’t as—again the last one I was almost in a meditative place. This one, I was for a little while but then I was more just, I was more aware for the majority of this float. But I was still super relaxed. I felt the crystals on my stomach and played with my hair again (laughs).

-*PARTICIPANT 15*

It went pretty well. I think it felt better than the float in that open pool.

-*PARTICIPANT 16*

It was good. Um, maybe a little bit longer than I wanted it to be. But overall I liked it better than the other float without the dome.

-*PARTICIPANT 17*

Overall the float was a good experience. Um…yeah! I actually really enjoyed it this time.

-*PARTICIPANT 18*

I guess it was a little distressing. Just because my chest started itching, and then it started burning. So I just couldn’t, like I felt relaxed in the beginning. I just couldn’t—after that I was just like I couldn’t relax.

-*PARTICIPANT 19*

It went pretty well. Felt like it took me a while to relax but after I relaxed it was great.

-*PARTICIPANT 20*

It went well. I was a lot more calm. Like I knew what to expect this time. And I just enjoyed it a lot more.

-*PARTICIPANT 22*

I thought it went really well. It was very relaxing and I enjoyed the experience.

-*PARTICIPANT 23*

**Enclosed pool float (Session #4)^^[[4]](#footnote-4)^^**

**Q- In general, how did the float go?**

This was definitely the best one. It was not unpleasant. It wasn’t like so pleasant that I would want to do it all the time, but it was definitely the most pleasant. And we were talking, I think one difference was that I came in just a really foul mood so right now I feel a lot better, um, and I really didn’t want to do the float at all, um, so maybe it was just because I was so dramatically negative and then when I finally did it, it ended up being really pleasant.

Q- So right now you’re feeling better?

1. A lot better, yeah. A lot more relaxed, um, not as um, more steady and not as emotional. Um, and a little more rational, which is unusual because normally I feel like, illogical.

-*PARTICIPANT 1*

Um, it was really good.

-*PARTICIPANT 2*

It went really well. I think it was my best float. I don’t know why, but I stayed awake the whole time and I feel more energetic afterwards.

-*PARTICIPANT 3*

Um, I felt it was um, relaxing. Um… this time my mind just for outside factors was a little more, um, chatterful. So I kind of really um, I couldn’t really let myself go.

Q- So what did you think about during the float? Positive thoughts? Negative thoughts? And if you had negative thoughts, how did you cope with them?

1. Um, I was kind of having some thoughts about while I was a patient here (laughs) and then I was having these, like, thoughts about being back in here. It was really weird. So some, like, things, memories that were popping up. And a little bit later I was thinking about what the rest of my day, what I needed to get done. And when I had the thoughts they were mainly, they were more neutral. They weren’t, yknow, positive or negative. So…

-SUBJECT 4

It was very relaxing.

-*PARTICIPANT 5*

It was my best one!

Q- You made it the longest!

1. I made it the longest and I enjoyed it the most as compared to the other ones.

Q- What made it the best compared to the other ones?

1. Um I think I was acclimated to the process by now, and, I don’t know…It was like, privy to relax. Like my attitude going into it was better than before.

-*PARTICIPANT 6*

It went great. It felt great.

-*PARTICIPANT 8*

Um, the float went really well. It was probably the…maybe I was more comfortable with the procedure and I’m gonna say that was probably the best one out of all of them so far. So I think it went very smoothly.

-*PARTICIPANT 9*

Um, I think it was a lot harder to relax this time than last time just cuz it was pretty warm, but like for the last maybe 30-40 minutes I got a lot more relaxed and it was more pleasant.

­*-PARTICIPANT 10*

It went well. It was good. It was pretty, it didn’t seem to take as long to get relaxed. I was a little more alert having the blood pressure and heart monitor on. And a little more focused on my heartrate than before. And, I felt like there were a few times where it would, my heart would start to race a little bit, or something, I don’t know if it was something I was thinking or feeling or what, or maybe it was the blood pressure, uh, that it would just bring my attention to that more, to my heart, and it made me feel, I don’t wanna say anxious, but maybe aware of it, more focused on it.

*-PARTICIPANT 11*

It was probably the second best one because I was sad that I felt sleeping couldn’t enjoy it (?) but it was enjoyable still even though I slept through part of it. But it was very relaxing.

-*PARTICIPANT 12*

It was good! It was probably my best of the 4.

-*PARTICIPANT 13*

The float went really well. It was really relaxing and it felt like the time went by fairly quickly.

-*PARTICIPANT 14*

Um, overall it went well. It was…I got a little restless probably the last 20-30 minutes. I had to pee and then I just kind of was like wanting to move a little bit. Um, but overall it was pretty good. I was able to relax for a good portion of it, and, um I did think about more I guess. Just about like, I prayed again and then I was thinking about this girl that came into this place that I volunteer yesterday and, um…I don’t know just stuff that’s going on with her and I was thinking about stuff with my kids and Christmas and stuff like that. So…but I was still really relaxed. I wasn’t like “oh my gosh I gotta get stuff done”. I was able to relax and just clearly think about stuff.

-*PARTICIPANT 15*

Well.

-*PARTICIPANT 16*

It was really good! It was probably the most relaxing float actually compared, even compared to last time. Yeah.

*-PARTICIPANT 17*

It went really well. Um, I would say this was my best float, maybe I’ve gotten used to it. Um, but for the most part it was really pleasant.

*-PARTICIPANT 18*

Um, the time passed really fast this time I thought. Like it did not feel like 90 minutes. Um, and, like initially the like, tickaderm, is that what it’s called?

*-PARTICIPANT 19*

It went well. I was able to stay in the whole time and I wasn’t super anxious or bored. I was pretty calm and relaxed.

*-PARTICIPANT 22*

It went really well. I felt relaxed most of the time. It was weird, I’d like—my mind was really awake but I think my body started falling asleep so I kept twitching, like a lot. I usually don’t do that all, but I probably twitched like 8 times.

*-PARTICIPANT 23*

**Session 1 (Chair-REST)^[[5]](#footnote-5)^**

**Q: What did you experience during the float?**

I experienced that panickiness, and then—I really feel like I thought, it was kind of obsessing about my body in the sense that, like, I couldn’t feel…like I felt kind of dissociated, like I couldn’t feel where the bounds of my body were if that makes any sense. Like I kind of morphed into the chair. And then I started thinking about, like, how cool that was and then I started thinking about my organs and like the implications my eating disorder has on my organs (mumbling).

Q- Did you have any positive experiences?

1. Yes! I think it was, like, a very, like, my mind tends to run 100 mph, so it was a good calming-down experience, but also like I was saying, I started thinking, like, not so much about my physical body as like the capacities that my body has. I don’t know I was thinking a lot about my kidneys…I don’t know (laughs).

-*PARTICIPANT 6*

Um… definitely at first, before I really had to go to the bathroom, I enjoyed the, uh, the darkened room, um, and laying back. So that was positive. On the other hand, negative experience: just kind of feeling, um… a little bit trapped and needing to go the bathroom really badly but not really wanting to, um, skew any of the results. So I was trying to maintain, um, thinking I could make it the full time, so I got a little bit anxious and a little panicky just, um, in the fact that I might not be able to complete the full 90 minutes as planned. So that was more of an anxious, nervous, negative experience.

-*PARTICIPANT 9*

Um, just felt very like relaxing and cozy (laughs). Just kind of funny to say. I think like the position of the chair, I think really helped too just because you were able to relax every single part of your body. And with the neck support too, I think that helped.

-*PARTICIPANT 13*

Um, like, I mean I, I got pretty tired. Cuz I had to like, it was hard to stay awake at some points.

-*PARTICIPANT 14*

Kinda just explained it in the last answer, but um… Really just, I was just relaxed and (laughs) it was nice! I was able to relax without my kids around. Yeah! I thought I was gonna be pretty bored and almost get agitated like I did for one of the last, like, in the MRI scanner. But it was actually just really relaxing and the time went by a lot faster.

1. No, not really negative. I mean my neck was to the side so my neck would get kind of stiff so I would just move it to the side. But other than that, no.

-*PARTICIPANT 15*

…….it was a lot colder than I wanted it to be. I might fall asleep (mumbling).

-*PARTICIPANT 16*

What did I experience? Um just a lot of relaxation.

-*PARTICIPANT 17*

Okay. Um I experienced…heaviness of my body. Um…I experienced, um, trying to stay still and be with myself, which I don’t do very often. Umm…I experienced tingling sensation, um…Felt kinda fuzzy in my body but really clear minded.

-*PARTICIPANT 18*

Um…I did notice my headache was worse at the beginning and then it kind of…it’s not as bad now. So that was a good thing.

-*PARTICIPANT 19*

Um…I mean it’s kinda interesting, you know I think I was trying to get my mind to calm down a little bit but I don’t know that it was really working. And then, I don’t, I don’t know. I don’t know maybe I fell asleep. I don’t know I was like “oh okay”, I just felt, I’m not very good with my words at putting what I feel, but… I don’t know I just didn’t feel as, um, rushed or something? I don’t know. Pressured? I don’t know. Sorry.

-*PARTICIPANT 20*

………….

-*PARTICIPANT 21*

Umm, I could feel my heartbeat like in my neck and I could hear it, kinda feel it in my ears. Um, just a feeling of like calmness and stillness kind of. I didn’t really feel, um—a lot times when I lay still I feel fidgety but this time I didn’t.

-*PARTICIPANT 22*

It was really—it didn’t take long for my eyes to adjust to the darkness. And then after a while I was like it’s almost too light in here, and that was weird. And after I think about 45 minutes I realized that I had to go to the bathroom and I waited as long as I could and then I was like I cant wait anymore.

-*PARTICIPANT 23*

NA

-All others

**Session 2 (Open pool float)^[[6]](#footnote-6)^**

**Q- What did you experience during the float?**

Um, I found myself the first, like 20 or 30 minutes, like I kind of mentioned dealing with, like, my head and neck and kind of a little worried and anxious about, um, that. So I, um, think that was a little bit of a negative experience. And I coped with that by, um, just waiting and kind of readjusting and realizing that, um, yknow my body was just tense, and, uh, so I. And the positive experiences, I felt like it was relaxing, um, I mean I’m always pretty tense so even floating I felt a bit on the anxious, tense side. But I just, I felt a little bit positive in that my mind wasn’t racing by so much. I think I felt like I kind of maybe was hitting a wall where I couldn’t quite get my brain moving quickly like it usually is. That was a positive thing, and a little bit distressing at the same time. But I would say mostly positive. It was a bit of a rest from—a forced rest bit—from excessive thoughts. It was just, yeah, I’m really used to overanalyzing everything and so when my mind isn’t constantly understanding and analyzing and figuring out what my surroundings are and how they effect my body and my mind, and kind of be able to anticipate, um, the future, like at this point floating, I just didn’t have a whole lot to, um, to think about and I think that’s a little distressing to me. Cuz I think generally I feel like whenever I can think through things it gives me a sense of confidence or a sense of control. So it was a little distressing just, um… But I would say that’s like a 10 out of 100 so, yknow, pretty minimal distress.

-*PARTICIPANT 9*

Most—99% positive. I just felt very comfortable in my body and very relaxed and it was a sensation that I’ve never felt before so that part was very new and exciting, which added to the positive aspect of it.

Q- Any negative?

1. Just my…looking at my fat belly (laughs).

Q- And how did you cope with that?

1. Uh I just kind of closed my eyes and drifted off, so…

-*PARTICIPANT 12*

Um well at first I felt like really heavy, and just like a lot of weight. Then eventually that kinda went away. But yeah that’s about it. I mean sometimes I could hear my breath or feel my heartrate a little bit, um, but other than that, no. Not a whole lot (laughs).

Q- Any specific positive experiences?

1. Um…it was kinda nice to feel weightless for a little while. And that kinda happened towards the end of it.

Q- Did you have any negative experiences?

1. Only kind of the first part where my neck was starting to hurt cuz I was holding my head up. But other than that no.

Q- How did you feel you coped with that?

1. Um eventually it just hurt so much that I just poked my head out.

-*PARTICIPANT 17*

Um I thought it was really cool how right when I got in I just laid back and floated to the top. I thought that was really neat. And I did at a few points, like I didn’t really even feel my body at all and then I kind of wiggled my toes and wiggled my fingers and I was like “oh yeah you’re still here”. I did get dizzy a few times, especially—because I opened my eyes even though it was pitch dark. Kind of made me dizzy so I just kept them closed a majority of the time. Um and then at the end when the music started playing I was like “okay I’m gonna move a little bit and just see what this feels like” so I was just playing with my hair, and stuff like that. It felt really cool. Kind of moving my arms around.

-*PARTICIPANT 15*

Well obviously physically I experienced like, feeling my breath and my heartbeat and then not being able to feel my body. Every once in a while I would kind of notice the water or I would notice like, the sound of the water or something maybe. And then I guess I felt, um, I don’t know just kind of attentive to that. Pretty at ease and relaxed and then near the end like I said a little anxious.

-*PARTICIPANT 14*

Um…I mean I had the neck tension that they described with like trying to hold your head up so I kept trying to relax it. And then I was more conscious of my breath then I’ve ever been. And then, yeah just feeling like the noises my stomach was making and my heart beating in my ears like I said. I don’t know I felt like I was super vigilant, like I felt like something was gonna happen at any moment. I don’t know just like laying there, I felt really vulnerable, so…

-*PARTICIPANT 6*

Um, I felt my heartbeat and breathing a lot more than I did when I was floating in the chair.

-*PARTICIPANT 16*

I could see colored spots like twice. Um, my eyes were open I think for the whole time. I can’t think of anything else. [Any positive experiences?] Um there were a few times where I would take a deep breath in. I guess I was able to relax my body for a short while. Like I didn’t feel as tense, like I made a real effort to like “okay relax your neck. I know I’ll still be floating. Relax this cuz it was hurting”. So I guess that was nice to be able to like relax for a pretty good time. (No other negative experiences)

-*PARTICIPANT 18*

Um, I don’t know, just, I felt like I was floating (laughs). I have a pool in my backyard so I just kind of felt like I was at my pool. Um…I did feel like the salt kind of crystallized on my face, which felt kind of weird. Yeah, I don’t know. I liked it.

-*PARTICIPANT 19*

I mean, I thought the whole thing was posi—I mean I guess I felt positive the whole time. After, I don’t know how long it takes me to get out of the “I’m stressed, I’m not doing right, I’m thinking about work”, I have no idea if that’s 5 minutes or 30 minutes. But then after, I guess I did have positive thoughts. I was like “well, I don’t even know if I’ll work today after this when I’m done.” Before I came here I was like “I have to go to work after this when I’m done”, which I really don’t have to. So I was laying there like “ohh maybe I don’t have to”, so for me that’s positive, like being able to make a decision. I don’t know I liked whenever I was stretching. The very end, when I couldn’t believe you could stretch and move and you didn’t sink. That was amazing! I really liked it. I really liked it. And I thought oh my gosh I wish this was something I could do all the time. Like I really feel like it helped me. I feel like it’s weird. Like I feel calm but I’m not sleepy. I’m not, I don’t know if I’m really energetic, but I’m just like good! So that was positive for me. I don’t know. I don’t know if that answered that your question. I told you I drove the grad student nuts last week probably.

-*PARTICIPANT 20*

Relaxation and I was a little cold.

-*PARTICIPANT 21*

I guess just the anxiety—like some anxiety, some calmness, and like when I was thinking about the ocean and stuff it was peaceful and my body felt pretty peaceful, but for some reason it switched. Umm, I didn’t like the feeling of like the salt drying on my skin. At very first before I put the Vaseline on it was painful, but otherwise it wasn’t painful. But like I didn’t like the feeling of the salt drying on my skin—like it felt gritty—but it wasn’t painful.

-*PARTICIPANT 22*

Just a very calm atmosphere, sort of refreshing.

-*PARTICIPANT 23*

Um, it didn’t…it felt shorter than it was. I didn’t think I’d been in there for almost an hour. And then I couldn’t, I was really surprised by how I couldn’t really feel myself moving at all. That was really weird to me! Yknow and then like, I kinda moved my arm in the water and I couldn’t really feel it. So that was weird, but it didn’t make me feel uncomfortable. I was just like “huh”.

-*PARTICIPANT 13*

NA

-All others

**Session 3 (First enclosed pool float)^[[7]](#footnote-7)^**

**Q: What did you experience during the float?**

I first thought… I first tried to pray the Rosary and I just gave up on that attempt because I really could not… I feel like I couldn’t concentrate, um… and so I just sort of let my mind go. I didn’t really think about anything, sort of had a blank mind. And then often, and then I just sort of started thinking when can we get out, when can I get out. So I tried to distract myself about thinking about other things, like reciting things that I have in my mind that are helpful for me to recite.

Q: Like what types of things?

A: Like a poem, um… that… yeah, a few months ago I realized I don’t really know… like I can’t really recite anything so I like learned it and that was very, um… helpful to be able to repeat, to repeat words. Um… so I have a poem I like to recite.

-*PARTICIPANT 1*

I had a song stuck in my head.

-*PARTICIPANT 2*

My mind just kind of wandered. Nothing in particular.

-*PARTICIPANT 3*

I was thinking about some of the things I had to do this afternoon, and about trying to let go of some body image issues that I’ve been trying to work out.

-*SUBJECT 4*

Well, in the beginning I was kind of like doing CBT things and then not really anything. Just like what I’m gonna do after this. And that’s it.

-*PARTICIPANT 5*

I don’t even know. *Laughs* Like, I’m just trying…I can’t really think. I can’t recall anything specific, like concentrating on anything.

Q- So no positive thoughts or negative thoughts?

1. No. It was just like being (laughs). Just floating.

-*PARTICIPANT 6*

I had negative thoughts about my body, but the more you start the float that I got, the less weight you felt, and that helped a lot, so I just tried to focus on the water and not my body. It was really hard at first, but, yeah… Yes I thought about my family. I thought about my schedule for just a minute and then I decided to just forget about it. So…

-*PARTICIPANT 8*

I had some positive thoughts in that I was glad not to be kind of, maybe worrying as quite as much as usual. Then I had some negative thoughts as far as body image goes, um, and I coped with those by… I think my mind just naturally kind of centered back to nothingness since it was so dark. So I don’t know if I really intentionally was able to put those thoughts out of my head but I did a little body checking, but I kind of was just able to let that go. It was just so dark and, and my mind was really sluggish.

-*PARTICIPANT 9*

I think that like in the middle I started thinking about homework and stuff that I had to do and I think that might be why I got a little bit like, “Oh god I have stuff to do!” But other than that, I kind of ended up blanking out at the beginning and end.

-*PARTICIPANT 10*

I thought about relaxing, trying to or to kinda, starting at my toes and trying to progressive relaxation. Make sure I was relaxing. I was thinking about all the different feelings and trying to figure out how, what words I could use to explain it to you. I did spend a lot of time doing that. I thought about my breathing a lot because it was, I could feel it and hear it quite a bit. But that was in a good way. Although I did, I forgot there were a couple times where I felt like I was kind of in tune with it, and then I wasn’t and I felt a bit startled, if that makes sense.

-*PARTICIPANT 11*

I thought about my boyfriend for a little bit. I thought about that my tattoos I’ve been wanting to get. I thought that I need to order some jelly plates for my art. I thought about how I need to finish my shopping.

-*PARTICIPANT 12*

I probably had a little more of just random thoughts this time. I think probably just because it was a Sunday afternoon and thinking about the week and stuff. But probably about halfway through I was able to kind of turn that off and just relax.

-*PARTICIPANT 13*

I thought about how my body felt in the water, and not being able to feel it, like I was just kind of floating around. I thought about just like the temperature and I guess I just thought a lot about how I felt in the pool.

-*PARTICIPANT 14*

Um…gosh I don’t really remember. It was just kind of fading thoughts. Nothing really—I did pray for part of it. I prayed a couple times actually. But like as far as…I didn’t…I don’t know. Just little flicking thoughts. Nothing that I can really remember for some reason. But I did—like I was just thinking about how it felt—like the way the water felt when I was moving around a little bit. I didn’t stay quite as still this time.

-*PARTICIPANT 15*

Nothing that stood out.

-*PARTICIPANT 16*

I really don’t know. Not a lot. I think I kind of fell asleep for a part of it.

-*PARTICIPANT 17*

I started off thinking about, um, well just kind of getting my surroundings, like checking the pool out. And then I did a lot of body checking. I was thinking like how much space I was taking up, how much water am I about to get in—things like that. So a lot of body at first and then, yeah I just kind of got most familiar with the stars, and like ping-ponged myself, so I was able to relax.

Q- And then you left the blue light on the whole time…

1. I did.

Q- So was that helpful?

1. Very helpful, yeah. I think just having the sense of being able to see where I am and like, I’m still here and I’m okay, and not having so much mind-body separation was helpful for me.

-*PARTICIPANT 18*

I just kind of, like, thought about what I did before, er, just kind of like leaving because like I really like the brain scan, so just thinking about all the people that I couldn’t, not scan. (?)

-*PARTICIPANT 19*

First trying to relax. Then sometimes I’d think about things I needed to do. Oh and then I was actually thinking about some people I needed to forgive. So…

-*PARTICIPANT 20*

I kind of tried to, like, just imagine, like, my dogs and just being at home, and what I was gonna do when I went home. Just like, stuff like that.

-*PARTICIPANT 22*

I thought this nightmare that I had last night. And then like floating in the pool kind of helped relax me and eventually bat that down.

-*PARTICIPANT 23*

**Session 4 (Second enclosed float pool)^^[[8]](#footnote-8)^^**

**Q- What did you experience during the float?**

Floating (laughs). Um the only thing that was kind of negative was when my neck got kind of stiff and my lower back got kind of stiff so I just bent my knees and put my feet on the bottom of the tank for a bit until that relaxed. Umm… I don’t know. The water was comfortable when I first got in but then it felt cooler towards the end. I felt a little chilly at the very end. I don’t know. That’s about it.

-*PARTICIPANT 15*

Um, positively I didn’t feel my outer body for a good amount of time so that felt really refreshing and I noticed that even my view of my physical appearance before and after changed a little bit. Um, negatively, it was just uh, I thought my armpits hurt. But I’m like “oh it’ll go away” and then my stomach was kind of gurgling a lot and I’m like “well…it’s okay”.

-*SUBJECT 4*

Mmm, my back quit hurting. I was in no pain. And that was the best part. I was in so much pain when I came in and the pain was just gone for the rest.

-*PARTICIPANT 12*

I was kinda like zoned out for a while. I don’t really know what happened. Time just kind of passed. I was just kind of there. It was kind of nice (laughs).

-*PARTICIPANT 6*

I could feel like, I could feel my breath and hear my breath and feel my heartbeat. And then I experienced not being able to feel my outer body for, not like the entire time but a majority of the time. And then just like being relaxed.

*-PARTICIPANT 14*

Sluggishness of thinking. My mind was kind of slow. Um… a little bit of mind wandering this time, yknow, kind of maybe it too… I don’t…I really, it might be one of my better floats because I didn’t have as much chatter. Yeah. I forget the rest of the question. (laughs)

*-PARTICIPANT 9*

………I don’t know. I was just more relaxed than normal.

*-PARTICIPANT 16*

Um, well I kinda felt a period where my legs kinda felt like they went away for a second. Um…but yeah.

*-PARTICIPANT 17*

Okay, um so I kind of said already that, like, there was a point where I really could not feel my body or, like, I wanted to turn the light on really bad cuz I…like one of my dreams or whatever was here turn the stars off. So I was like “I need to turn the light on” um, and I couldn’t move my arm, like I kept thinking that I had turned the light on and my eyes were opened, I was fully aware that the lights were not on. So that went on for a while, where like my eyelids would close but I wouldn’t realize they were closed and I was like “she turned the stars off. Like why did she turn the stars off?” Um, so and when I did hear the knocking on the door, my response was really to, cuz I couldn’t—I knew I couldn’t get up. I was like, I can’t do anything and so finally I was like 1, 2, 3 go and then poked my head up and then I thought you were there but you weren’t so that was like my biggest experience this time, was like my mind was there. Like I was…I didn’t feel like I was sleeping but I just really could not feel my body very much, so. It was a strange feeling.

*-PARTICIPANT 18*

Um…again I thought the temperature was good. Er I mean I do wish it was a little warmer but I didn’t feel like a big difference between the air and the water.

*-PARTICIPANT 19*

Um, nothing really, just kind of a calm feeling. I fell asleep for maybe a fifth of it or less. Something like that. But yeah.

*-PARTICIPANT 22*

Um, overall, a sense of calm, refreshing.

*-PARTICIPANT 23*

**Session 1 (Chair-REST)^[[9]](#footnote-9)^**

**Q: Did you have any positive experiences during the float?**

Yes! I think it was, like, a very, like, my mind tends to run 100 mph, so it was a good calming-down experience, but also like I was saying, I started thinking, like, not so much about my physical body as like the capacities that my body has. I don’t know I was thinking a lot about my kidneys…I don’t know (laughs).

-*PARTICIPANT 6*

Just was able to think more rationally. [What do you mean by that?] Um, instead of letting my anxiety kind of rule my thoughts and everything. Cuz I think about, yknow stuff I’ve been told over the years, and trained somewhat to think, um it was much easier to take that route, so…

-*PARTICIPANT 13*

I felt pretty relaxed coming into it, but I think it is a positive that I didn’t feel like—that I don’t feel like as relaxed now. Like I wasn’t really anxious during the time. So I’d say that’s a positive.

-*PARTICIPANT 19*

I’d say being relaxed was pretty positive.

-*PARTICIPANT 14*

I didn’t have any negative experiences, but it was, um, and nothing like positive that I can…I mean nothing bothered me. It’s not that. But…one time I felt like maybe just for like a second or two I felt that maybe I just didn’t feel my outer body. It was not long at all. Just very few seconds. [And would you say that was positive, negative, or neutral?] Positive!

-*PARTICIPANT 20*

I think floating in the chair was positive. [And what made it positive?] It was relaxing.

-*PARTICIPANT 21*

Just like the relaxation.

-*PARTICIPANT 16*

Yeah I mean I think overall the entire thing was really positive.

-*PARTICIPANT 17*

It was really relaxing.

-*PARTICIPANT 23*

NA

-All others

**Session 2 (Open pool float)^[[10]](#footnote-10)^**

**Q: Did you have any positive experiences during the float?**

Um…it was kinda nice to feel weightless for a little while. And that kinda happened towards the end of it.

-*PARTICIPANT 17*

Yeah I would say just the, it was a neat experience being able to not feel some of my sensations.

-*PARTICIPANT 14*

None that I haven’t mentioned.

-*PARTICIPANT 19*

Umm, it was kinda cool to see what it’s like to be like, buoyant like that. (laughs)

-*PARTICIPANT 6*

Just like the float, like, overall, was positive. [What made it positive?] It was relaxing.

-*PARTICIPANT 16*

It was—a positive experience was that it was relaxing.

-*PARTICIPANT 21*

Um, I feel like that 20% that I felt calm, it was positive. I felt like, mindful and relaxed and I thought that it was cool that I was just floating. So I guess I thought it was cool how it worked.

-*PARTICIPANT 22*

NA

-All others

**Session 3 (First enclosed pool float)^[[11]](#footnote-11)^**

**Q: Did you have any positive experiences during the float?**

Pleasant aspects, uh… um… I think it’s fun to experience just lying on the surface of water, but… it was pleasant to have a quiet still room. But, I think that… I mean you could add this in later, but I think that since I don’t have like a very stimulated life, cause I live alone, and… I don’t have children… it’s not this huge drastic change from my normal, regular life. My life isn’t very loud or very active, intentionally; I did that on purpose.

-*PARTICIPANT 1*

It was pretty neutral.

-*PARTICIPANT 2*

Yes. It was very relaxing.

-*PARTICIPANT 3*

Just relaxation and then in general it relieved muscle tension and pain.

-*SUBJECT 4*

Yeah, I got comfortable. It was like I settled in a lot quicker today. So that was good.

-*PARTICIPANT 5*

Yeah I made it longer than I did last time, so I’m pretty proud of myself. So that was pretty good.

-*PARTICIPANT 6*

Oh definitely! Just the peace and quiet and the weightlessness was a nice break for me and just being able to not stay focused on my body image and my body was really nice for a change.

-*PARTICIPANT 8*

I enjoyed the feeling of weightlessness. Kind of when I, I tried to stay as still as possible, but when I did move my arms at all, they just kind of readjusted to some neutral/natural position. So that was kind of how my experience was as far as the float goes.

-*PARTICIPANT 9*

Oh yeah. The rest of the float was great. Really relaxing.

-*PARTICIPANT 10*

So far it was telling you a minute ago that it was like this last time too, but I wasn’t really sure how to describe it. Related to my eating disorder, I’ve always had an issue with my stomach and then uncomfortable with it, and it’s always, on some level, in my awareness. And, when I was floating, it was more of a neutral feeling that it, I almost felt like my body felt stretched and a little bit disconnected from my limbs at times, and maybe even from my stomach. But it was a little bit like it was pressure on it, but not at all uncomfortable. It felt, just normal, I guess would be a good word. It felt normal and okay.

-*PARTICIPANT 11*

The atmosphere was awesome. It was so beautiful and I did like being in the water and I was able to relax for 20 minutes or so.

-*PARTICIPANT 12*

Just really the relaxation. So obviously that 50 minutes is kind of a good magic number for me I guess.

-*PARTICIPANT 13*

Yeah I thought it felt kind of cool, like, “Oh I can’t really feel my body! Oh I can’t really see anything.” Yeah, I was kind of losing the sensation.

-*PARTICIPANT 14*

Really just the—I don’t know, the water feels really good when you move, just the way it passes over the skin. I did, like I didn’t feel myself there for a while. Just kind of didn’t feel much of anything. But, I don’t know. I think the whole experience is pretty positive. I did have to pee towards the end so that wasn’t real pleasant, but it wasn’t bad enough where I needed to get out.

-*PARTICIPANT 15*

I guess you can count, like, at one point, because this didn’t really happen last float, I couldn’t really tell where my skin stopped and the water started. I guess that’s positive. It was kinda cool.

-*PARTICIPANT 16*

Yeah my body was just really relaxed, I think, the most it’s been throughout this process.

-*PARTICIPANT 17*

I feel like my mind just kind of, kind of went blank. I was really enjoying the sensation of floating so I guess that’s a thought. Of, like, almost feeling weightless, so I thought about that for a while.

-*PARTICIPANT 18*

I did feel like, like the past few days I haven’t really had any body image distress, and I think this is the only thing I’ve done differently. So I think it has helped in that regard. [And] I did think that the like domed part helped with the temperature.

-*PARTICIPANT 19*

Well yeah I mean I like not feeling like—it’s kind of a “you don’t really feel your extremities, you don’t feel your body, you don’t feel your weight.” But you’re not floating going crazy on drugs, which I’ve never done drugs. But it’s just like a good, it’s kind of like a high, but, I don’t know… I liked it! I liked floating. I liked feeling like you’re just laying on air.

-*PARTICIPANT 20*

The water and like the air was a really good temperature, and it was just very comfortable, and I guess for some reason I liked the dome more than the open one. I don’t really know why. I guess just the feeling that it was enclosed felt good I guess. I don’t really know. Because I had the light off so it wasn’t like I could really tell. I guess it was just knowing it. But yeah.

-*PARTICIPANT 22*

It didn’t sting! I have a little cut on my finger but I put Vaseline on and it didn’t hurt. I was worried about it.

-*PARTICIPANT 23*

**Session 4 (Second enclosed pool float)^[[12]](#footnote-12)^**

**Q: Did you have any positive experiences during the float?**

It was just nice to get away and like, I don’t know, a secluded atmosphere where nobody’s gonna bother you, and just really be alone because, you never really are, so…Just taking time off I guess.

*-PARTICIPANT 12*

Yeah! I wasn’t like, I felt like my mind shut off more this float than it did the other ones. Um, so that was, that like made it easier instead of thinking about more I was doing the whole time, which isn’t relaxing at all.

*-PARTICIPANT 6*

Um, I probably felt the most—I think with all of them I always kind of felt like I was literally floating and you couldn’t feel the rest of your body, but probably more so this one. And it was the first time I was able to feel my heartbeat, and, um, like I could hear my digestive system a lot better too (laughs).

*-PARTICIPANT 13*

Um, yeah I would say that being relaxed and losing the sensations.

*-PARTICIPANT 14*

Eh.

*-PARTICIPANT 16*

I felt like the temperature of the air and the water were a lot closer this time. I don’t know if they were changed or not but they felt like they were the same.

*-PARTICIPANT 17*

I felt like, like that was positive. Um, I thought it was cool that I wasn’t, I didn’t feel bad in my body. I just didn’t feel my body at all. So I thought that that was cool.

*-PARTICIPANT 18*

Um, yeah I liked—well I mean the light was neutral but like the feeling of my body and the water, that was positive.

*-PARTICIPANT 19*

Um, I felt really calm so that was good. And, um, the temperature was really good. It felt pleasant.

*-PARTICIPANT 22*

The calmness was nice.

*-PARTICIPANT 23*

NA

*-All others*

**Session 1 (Chair-REST)^[[13]](#footnote-13)^**

**Q: Did you have any negative experiences during the float?**

Um…No I got a little cold, but, that’s it.

-*PARTICIPANT 18*

*shakes head no*

-*PARTICIPANT 13*

I did feel like I was kind of fidgeting a lot. I think it was cuz I was trying to like warm myself up a little bit. [How did you cope with that?] Um, I just tried to, like, find the position that was most comfortable and just kind of stick with that.

-*PARTICIPANT 19*

I just got kinda cold and tired. [And how did you cope with those experiences?] I just kinda pushed through ‘em I guess (laughs).

-*PARTICIPANT 14*

No, not really negative. I mean my neck was to the side so my neck would get kind of stiff so I would just move it to the side. But other than that, no.

-*PARTICIPANT 15*

*shakes head no*

-*PARTICIPANT 20*

No.

-*PARTICIPANT 21*

Aside from the thoughts, no.

-*PARTICIPANT 17*

No.

-*PARTICIPANT 22*

No.

-*PARTICIPANT 23*

NA

-All others

**Session 2 (Open pool float)^[[14]](#footnote-14)^**

**Q: Did you have any negative experiences during the float?**

Just my…looking at my fat belly (laughs). Uh I just kind of closed my eyes and drifted off, so…

-*PARTICIPANT 12*

Only kind of the first part where my neck was starting to hurt cuz I was holding my head up. But other than that no. Um eventually it just hurt so much that I just poked my head out.

-*PARTICIPANT 17*

Not really besides just wanting to get out at the end.

-*PARTICIPANT 14*

Um, I probably got some of the water in my mouth and it tasted really bitter. I just kinda got some spit and just…
-*PARTICIPANT 19*

Like just talking myself down like I already said.

-*PARTICIPANT 6*

No.

-*PARTICIPANT 16*

Um when I turned the light off I did not like that. No. I felt like, I just didn’t like not seeing where I was and then being in water. It just felt too not in control and, yeah. Like I was just in some random ocean floating away (laughs).

-*PARTICIPANT 13*

[So how did you cope with being cold?] Umm…I would like put my hand down, cuz like at the bottom of the pool it was warmer.

-*PARTICIPANT 21*

Just tried to think about other things.

-*PARTICIPANT 22*

NA

-All others

**Session 3 (First enclosed pool float)^[[15]](#footnote-15)^**

**Q: Did you have any negative experiences during the float?**

Unpleasant aspect of the float…uh… um… okay. Air felt heavy on me. I could breathe, but I prefer cooler air. Unpleasant… I didn’t… I guess I didn’t know what the point of it was, like why was I there and what was I doing. And I thought, okay I am here to relax and it wasn’t compelling enough to make me stay. A few times I thought I was going to get out. Unpleasant… I… guess I don’t… really felt warm. Yeah, it kind of felt heavy. I was going to say it’s pleasant to lie down. I guess it just overall felt too warm… yeah.

-*PARTICIPANT 1*

I got a little bored at times, but not as much as before.

-*PARTICIPANT 2*

No.

-*PARTICIPANT 3*

Just, I had a very slight pain spasm in my left knee and butt. Lower butt area. But it wasn’t very long.

-*SUBJECT 4*

*shakes head no*

-*PARTICIPANT 5*

I kept on twitching. Like I’d be totally calm and then my body would like twitch or one of my limbs would twitch and then that kind of aroused me.

-*PARTICIPANT 6*

No. There were no negatives.

-*PARTICIPANT 8*

Um, no. Not really. It was all very pleasant.

-*PARTICIPANT 9*

I think that I just like normally, the last time I floated I was like, a little bit antsy at the beginning of the float and this time that happened randomly in the middle, but it passed so…

-*PARTICIPANT 10*

A little bit discomfort in my neck but not nearly as much as last time. I had to go to the bathroom at the beginning but that was resolved, so it wasn’t uncomfortable for long, so, no.

-*PARTICIPANT 11*

**Session 4 (Second enclosed pool float)^[[16]](#footnote-16)^**

**Q: Did you have any negative experiences during the float?**

No.

-*PARTICIPANT 15*

No.

*-PARTICIPANT 6*

Um, just kind of anxiety about the future. I think I probably did play over some, kind of, some kind of going through a job transition right now, and, um, well, I don’t have a job right now so, as of pretty recently, so those are kind of some negative thoughts. Just what the future holds. It wasn’t really terrible or frightening, but it was kind of depressing. A little depressing.

Q- How did you cope with the negative thoughts?

1. I think I coped—a lot of times I seem to cope, like new experiences or thinking about things in different…reframing I guess as therapists like to say. So I think I coped with them kind of by realizing that I was in kind of a different, I was experiencing something pretty unique and that kind of is distracting from negative thoughts. Just the simple fact of, um, of experiencing something new and kind of, um, just seeing, wanting to do everything right and, um just, experience something new. (laughs)

*-PARTICIPANT 9*

Um, body image thoughts yeah.

Q- How did you cope with those thoughts?

1. I just figured “well I could lose weight so it’s okay”.

*-PARTICIPANT 12*

Just near the end when I was ready to get out.

*-PARTICIPANT 14*

Nope.

*-PARTICIPANT 16*

Um, body stuff was pretty, um, I mean it just kept coming up. I guess it was negative but I would redirect myself. Um…other than that…no.

*-PARTICIPANT 18*

Um I guess I thought a little bit about, why did I come here? Like, sometimes I deal--I’m like I shouldn’t have come here, like I didn’t need to come here-- I mean I needed to come here, but—

*-PARTICIPANT 19*

Um, a little bit just about like body image. But other than that….

*-PARTICIPANT 22*

NA

*-PARTICIPANT 23*

NA

*-PARTICIPANT 17*

NA

*-PARTICIPANT 1*

NA

­*-PARTICIPANT 2*

NA

-*PARTICIPANT 3*

NA

-*SUBJECT 4*

NA

-*PARTICIPANT 5*

NA

-*PARTICIPANT 8*

NA

-*PARTICIPANT 10*

NA

-*PARTICIPANT 11*

NA

-*PARTICIPANT 13*

1. Note: subsequent prompting questions are identified via the -Q prefix, with answers denoted by the -A prefix. [↑](#footnote-ref-1)
2. Note: subsequent prompting questions are identified via the -Q prefix, with answers denoted by the -A prefix. Missing responses = no answer provided. NA = participant stated not applicable. [↑](#footnote-ref-2)
3. Note: subsequent prompting questions are identified via the -Q prefix, with answers denoted by the -A prefix. Missing responses = no answer provided. NA = participant stated not applicable. [↑](#footnote-ref-3)
4. Note: subsequent prompting questions are identified via the -Q prefix, with answers denoted by the -A prefix. [↑](#footnote-ref-4)
5. Note: subsequent prompting questions are identified via the -Q prefix, with answers denoted by the -A prefix. Missing responses = no answer provided. NA = participant stated not applicable. [↑](#footnote-ref-5)
6. Note: subsequent prompting questions are identified via the -Q prefix, with answers denoted by the -A prefix. Missing responses = no answer provided. NA = participant stated not applicable. [↑](#footnote-ref-6)
7. Note: subsequent prompting questions are identified via the -Q prefix, with answers denoted by the -A prefix. Missing responses = no answer provided. NA = participant stated not applicable. [↑](#footnote-ref-7)
8. Note: subsequent prompting questions are identified via the -Q prefix, with answers denoted by the -A prefix. Missing responses = no answer provided. NA = participant stated not applicable. [↑](#footnote-ref-8)
9. Note: subsequent prompting questions are identified via the -Q prefix, with answers denoted by the -A prefix. Missing responses = no answer provided. NA = participant stated not applicable. [↑](#footnote-ref-9)
10. Note: subsequent prompting questions are identified via the -Q prefix, with answers denoted by the -A prefix. Missing responses = no answer provided. NA = participant stated not applicable. [↑](#footnote-ref-10)
11. Note: subsequent prompting questions are identified via the -Q prefix, with answers denoted by the -A prefix. Missing responses = no answer provided. NA = participant stated not applicable. [↑](#footnote-ref-11)
12. Note: subsequent prompting questions are identified via the -Q prefix, with answers denoted by the -A prefix. Missing responses = no answer provided. NA = participant stated not applicable. [↑](#footnote-ref-12)
13. Note: subsequent prompting questions are identified via the -Q prefix, with answers denoted by the -A prefix. Missing responses = no answer provided. NA = participant stated not applicable. [↑](#footnote-ref-13)
14. Note: subsequent prompting questions are identified via the -Q prefix, with answers denoted by the -A prefix. Missing responses = no answer provided. NA = participant stated not applicable. [↑](#footnote-ref-14)
15. Note: subsequent prompting questions are identified via the -Q prefix, with answers denoted by the -A prefix. Missing responses = no answer provided. NA = participant stated not applicable. [↑](#footnote-ref-15)
16. Note: subsequent prompting questions are identified via the -Q prefix, with answers denoted by the -A prefix. Missing responses = no answer provided. NA = participant stated not applicable. [↑](#footnote-ref-16)
