# Supplementary material for: Reduced Environmental Stimulation in Anorexia Nervosa: An Early-Phase Clinical Trial
Source: Front Psychol. 2020 Oct 6;11:567499. doi: 10.3389/fpsyg.2020.567499 (PMC7573249; doi:10.3389/fpsyg.2020.567499)
Supplement: Supplementary file 2 [file Data_Sheet_2.docx]

**Reduced environmental stimulation in anorexia nervosa:**

**an early phase clinical trial**

***Supplemental Information***

**Table of Contents**

Supplemental Instructions – Page 2

Supplemental Table – Page 10

Supplemental Figures – Page 12

**Pre session instructions – chair REST (session #1)**

This will be your first float, out of four. Today you will be floating in the chair. Today we will be recording your blood pressure, heart and breathing rate during the float.

A few things to remember: while floating, try to remain still. It’s okay if you move, but just try your best not to move too much. Also, try your best not to fall asleep. We realize that you might fall asleep on occasion, but it’s important to keep in mind that our study is focused on what happens to the brain while you are awake. Try your best to float with the lights off. You are free to turn the lights on whenever you need them, but please try your best to float with the lights off.

You can float for as long as you want, for up to 90 minutes. You are always free to stop floating at any time. Don’t worry, we’ll be sure to turn on some music at the end of the session to let you know that the float is almost over. Please remain floating with the lights off until the music stops, at which point we will turn on the lights for you.

After the float is over, you can put on your bathrobe and we will help take off your sensors before showering. We don’t want to interfere with your float experience before we collect your ratings, so we will try to minimize our dialogue until after we collect your ratings.

After you have showered, all you need to do is come back to the lounge area and take a seat on the couch. We will collect your ratings and interview you about your experience.

Before you start floating, we will be collecting 3 minutes of baseline measurements. There is nothing for you to do except remain quiet and still while you stand next to the chair. I will let you know when the baseline period ends, at which point you can begin your float session.

Do you have any questions? Are you ready to begin?

**Pre session instructions – open pool float (session #2)**

This will be your second float, out of four. Today you will be floating in the open pool. Today we will be recording your blood pressure, heart and breathing rate during the float.

A few things to remember: while floating, try to remain still. It’s okay if you move, but just try your best not to move too much. Also, try your best not to fall asleep. We realize that you might fall asleep on occasion, but it’s important to keep in mind that our study is focused on what happens to the brain while you are awake. Try your best to float with the lights off. You are free to turn the lights on whenever you need them, but please try your best to float with the lights off.

You can float for as long as you want, for up to 90 minutes. You are always free to stop floating at any time. Don’t worry, we’ll be sure to turn on some music at the end of the session to let you know that the float is almost over. Please remain floating with the lights off until the music stops, at which point we will turn on the lights for you.

After the float is over, you can put on your bathrobe and we will help take off your sensors before showering. We don’t want to interfere with your float experience before we collect your ratings, so we will try to minimize our dialogue until after we collect your ratings.

After you have showered, all you need to do is come back to the lounge area and take a seat on the couch. We will collect your ratings and interview you about your experience.

Before you start floating, we will be collecting 3 minutes of baseline measurements. There is nothing for you to do except remain quiet and still while you stand next to the pool. I will let you know when the baseline period ends, at which point you can begin your float session.

Do you have any questions? Are you ready to begin?

**Pre session instructions – enclosed pool float (session #3)**

This will be your third float, out of four. Today you will be floating in the domed pool. Today we will not be measuring your blood pressure, heart or breathing rate during the float.

A few things to remember: while floating, try to remain still. It’s okay if you move, but just try your best not to move too much. Also, try your best not to fall asleep. We realize that you might fall asleep on occasion, but it’s important to keep in mind that our study is focused on what happens to the brain while you are awake. Try your best to float with the lights off. You are free to turn the lights on whenever you need them, but please try your best to float with the lights off.

For this float, we would like you to float for the full 90 minutes. However, you are always free to stop floating at any time if you want to get out early. We would also like you to float with the lights off. Don’t worry, we’ll be sure to turn on some music at the end of the session to let you know that the float is almost over. Please remain floating with the lights off until the music stops, at which point we will turn on the lights for you.

After the float is over you can immediately begin showering. We don’t want to interfere with your float experience before we collect your ratings, so we will try to minimize our dialogue until after we collect your ratings.

After you have showered, all you need to do is come back to the lounge area and take a seat on the couch. We will collect your ratings and interview you about your experience.

Before you start floating, we will be collecting 3 minutes of baseline measurements. There is nothing for you to do except remain quiet and still while you stand next to the pool. I will let you know when the baseline period ends, at which point you can begin your float session.

Do you have any questions? Are you ready to begin?

**Pre session instructions – enclosed pool float (session #4)**

This will be your fourth and final float, out of four. Today you will be floating in the domed pool. Today we will be recording your blood pressure, heart and breathing rate during the float.

A few things to remember: while floating, try to remain still. It’s okay if you move, but just try your best not to move too much. Also, try your best not to fall asleep. We realize that you might fall asleep on occasion, but it’s important to keep in mind that our study is focused on what happens to the brain while you are awake. Try your best to float with the lights off. You are free to turn the lights on whenever you need them, but please try your best to float with the lights off.

For this float, we would like you to float for the full 90 minutes. However, you are always free to stop floating at any time if you want to get out early. We would also like you to float with the lights off. Don’t worry, we’ll be sure to turn on some music at the end of the session to let you know that the float is almost over. Please remain floating with the lights off until the music stops, at which point we will turn on the lights for you.

After the float is over, you can put on your bathrobe and we will help take off your sensors before showering. We don’t want to interfere with your float experience before we collect your ratings, so we will try to minimize our dialogue until after we collect your ratings.

After you have showered, all you need to do is come back to the lounge area and take a seat on the couch. We will collect your ratings and interview you about your experience.

Before you start floating, we will be collecting 3 minutes of baseline measurements. There is nothing for you to do except remain quiet and still while you stand next to the pool. I will let you know when the baseline period ends, at which point you can begin your float session.

Do you have any questions? Are you ready to begin?

**Supplemental Table 1. Individual effect sizes and 95% confidence interval estimates for all secondary outcome measures.**

| **Measure** | **Session** | **Effect size** | **Lower boundary** | **Upper boundary** |
| --- | --- | --- | --- | --- |
| Negative Affect | Chair_1 | -1.13 | -1.78 | -0.74 |
| Fatigue | Chair_1 | -0.47 | -0.96 | -0.08 |
| State Anxiety | Chair_1 | -1.2 | -1.98 | -0.86 |
| Stress | Chair_1 | -1.28 | -1.86 | -0.94 |
| Refreshed | Chair_1 | 1.33 | 0.88 | 2.19 |
| Serenity | Chair_1 | 1.08 | 0.65 | 1.85 |
| Relaxation | Chair_1 | 1.22 | 0.84 | 1.86 |
| Energy | Chair_1 | 1.13 | 0.78 | 1.7 |
| Happiness | Chair_1 | 0.4 | 0.02 | 0.9 |
| Positive Affect | Chair_1 | 0.06 | -0.4 | 0.47 |
| Breath Intensity | Chair_1 | 0.08 | -0.4 | 0.47 |
| Heartbeat Intensity | Chair_1 | 0.38 | -0.02 | 0.87 |
| Stomach Intensity | Chair_1 | -0.33 | -0.81 | 0.07 |
| Systolic BP | Chair_1 | 0.45 | 0.03 | 1.03 |
| Diastolic BP | Chair_1 | 0.74 | 0.27 | 1.52 |
| Heart Rate | Chair_1 | 0.03 | -0.48 | 0.5 |
| Current Body Size | Chair_1 | -0.5 | -0.86 | -0.17 |
| Ideal Body Size | Chair_1 | 0.35 | 0 | 0.71 |
| Body Dissatisfaction | Chair_1 | -0.67 | -1.14 | -0.35 |
| BISS | Chair_1 | 0.71 | 0.37 | 1.14 |
| Negative Affect | Pool_1 | -0.59 | -1.55 | -0.13 |
| Fatigue | Pool_1 | -0.65 | -1.05 | -0.33 |
| State Anxiety | Pool_1 | -1.12 | -2.22 | -0.61 |
| Stress | Pool_1 | -1.3 | -1.94 | -0.95 |
| Refreshed | Pool_1 | 1.09 | 0.6 | 2.05 |
| Serenity | Pool_1 | 1.23 | 0.74 | 2.13 |
| Relaxation | Pool_1 | 1.13 | 0.61 | 2.21 |
| Energy | Pool_1 | 0.56 | 0.16 | 1.12 |
| Happiness | Pool_1 | 0.17 | -0.25 | 0.64 |
| Positive Affect | Pool_1 | 0.14 | -0.28 | 0.57 |
| Breath Intensity | Pool_1 | 0.34 | -0.07 | 0.86 |
| Heartbeat Intensity | Pool_1 | 0.44 | 0.02 | 1 |
| Stomach Intensity | Pool_1 | -0.01 | -0.46 | 0.42 |
| Systolic BP | Pool_1 | -0.08 | -0.47 | 0.5 |
| Diastolic BP | Pool_1 | -0.38 | -0.76 | 0 |
| Heart Rate | Pool_1 | 0.6 | 0.12 | 1.43 |
| Current Body Size | Pool_1 | -0.32 | -0.86 | 0.08 |
| Ideal Body Size | Pool_1 | 0.12 | -0.3 | 0.45 |
| Body Dissatisfaction | Pool_1 | -0.3 | -0.73 | 0.11 |
| BISS | Pool_1 | 0.48 | 0.11 | 0.91 |
| Negative Affect | Pool_2 | -0.53 | -1.15 | -0.1 |
| Fatigue | Pool_2 | -0.43 | -0.96 | -0.01 |
| State Anxiety | Pool_2 | -0.99 | -1.8 | -0.5 |
| Stress | Pool_2 | -1.26 | -2.03 | -0.81 |
| Refreshed | Pool_2 | 1.26 | 0.75 | 2.23 |
| Serenity | Pool_2 | 1.01 | 0.61 | 1.68 |
| Relaxation | Pool_2 | 1.43 | 0.91 | 2.55 |
| Energy | Pool_2 | 0.61 | 0.17 | 1.27 |
| Happiness | Pool_2 | 0.26 | -0.16 | 0.93 |
| Positive Affect | Pool_2 | 0.34 | -0.08 | 0.88 |
| Breath Intensity | Pool_2 | 0.49 | 0.07 | 1.06 |
| Heartbeat Intensity | Pool_2 | 0.33 | -0.09 | 0.98 |
| Stomach Intensity | Pool_2 | -0.11 | -0.5 | 0.39 |
| Current Body Size | Pool_2 | -0.46 | -1.24 | 0 |
| Ideal Body Size | Pool_2 | 0.12 | -0.32 | 0.47 |
| Body Dissatisfaction | Pool_2 | -0.41 | -1.01 | 0 |
| BISS | Pool_2 | 0.77 | 0.51 | 1.15 |
| Negative Affect | Pool_3 | -0.91 | -1.32 | -0.66 |
| Fatigue | Pool_3 | -0.43 | -1.07 | -0.01 |
| State Anxiety | Pool_3 | -1.42 | -2.1 | -1.02 |
| Stress | Pool_3 | -1.34 | -2.12 | -0.91 |
| Refreshed | Pool_3 | 1.58 | 1.03 | 2.61 |
| Serenity | Pool_3 | 1.67 | 1.28 | 2.41 |
| Relaxation | Pool_3 | 1.64 | 1.21 | 2.59 |
| Energy | Pool_3 | 0.76 | 0.32 | 1.54 |
| Happiness | Pool_3 | 0.59 | 0.24 | 1 |
| Positive Affect | Pool_3 | 0.69 | 0.28 | 1.29 |
| Breath Intensity | Pool_3 | 0.35 | -0.08 | 0.83 |
| Heartbeat Intensity | Pool_3 | 0.27 | -0.15 | 0.8 |
| Stomach Intensity | Pool_3 | 0.09 | -0.34 | 0.57 |
| Systolic BP | Pool_3 | -0.24 | -0.98 | 0.17 |
| Diastolic BP | Pool_3 | -0.08 | -0.56 | 0.37 |
| Heart Rate | Pool_3 | 0.27 | -0.17 | 1.03 |
| Current Body Size | Pool_3 | -0.79 | -1.24 | -0.51 |
| Ideal Body Size | Pool_3 | 0.47 | 0.22 | 0.77 |
| Body Dissatisfaction | Pool_3 | -0.91 | -1.35 | -0.63 |
| BISS | Pool_3 | 0.77 | 0.36 | 1.35 |

**
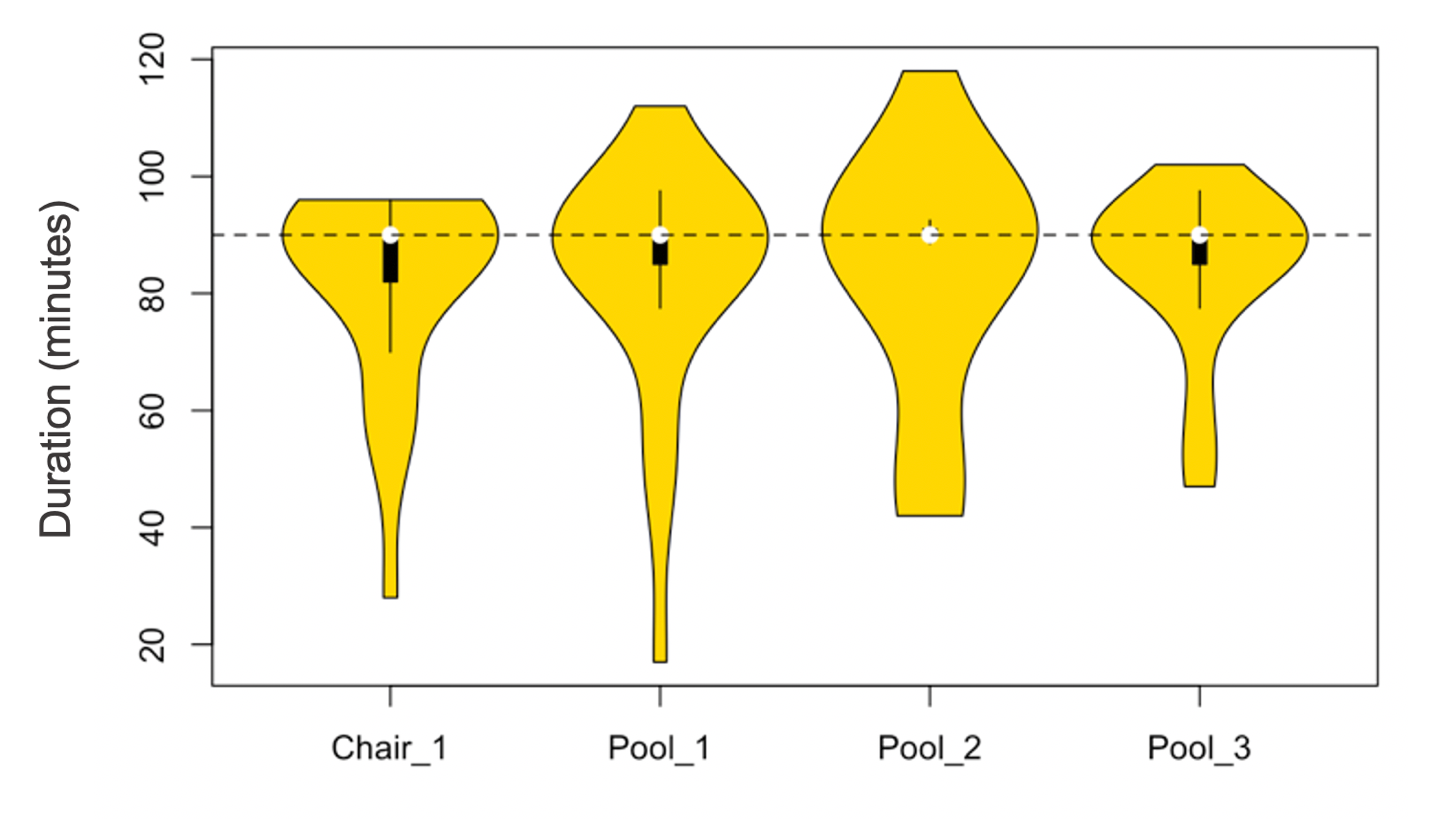
**

**Supplemental Figure 1. Session duration.** Violin plot showing the distribution of float durations for each float session. The white circles indicate the maximum duration of the float session (90 minutes). Most individuals floated for the entire 90 minutes.

**
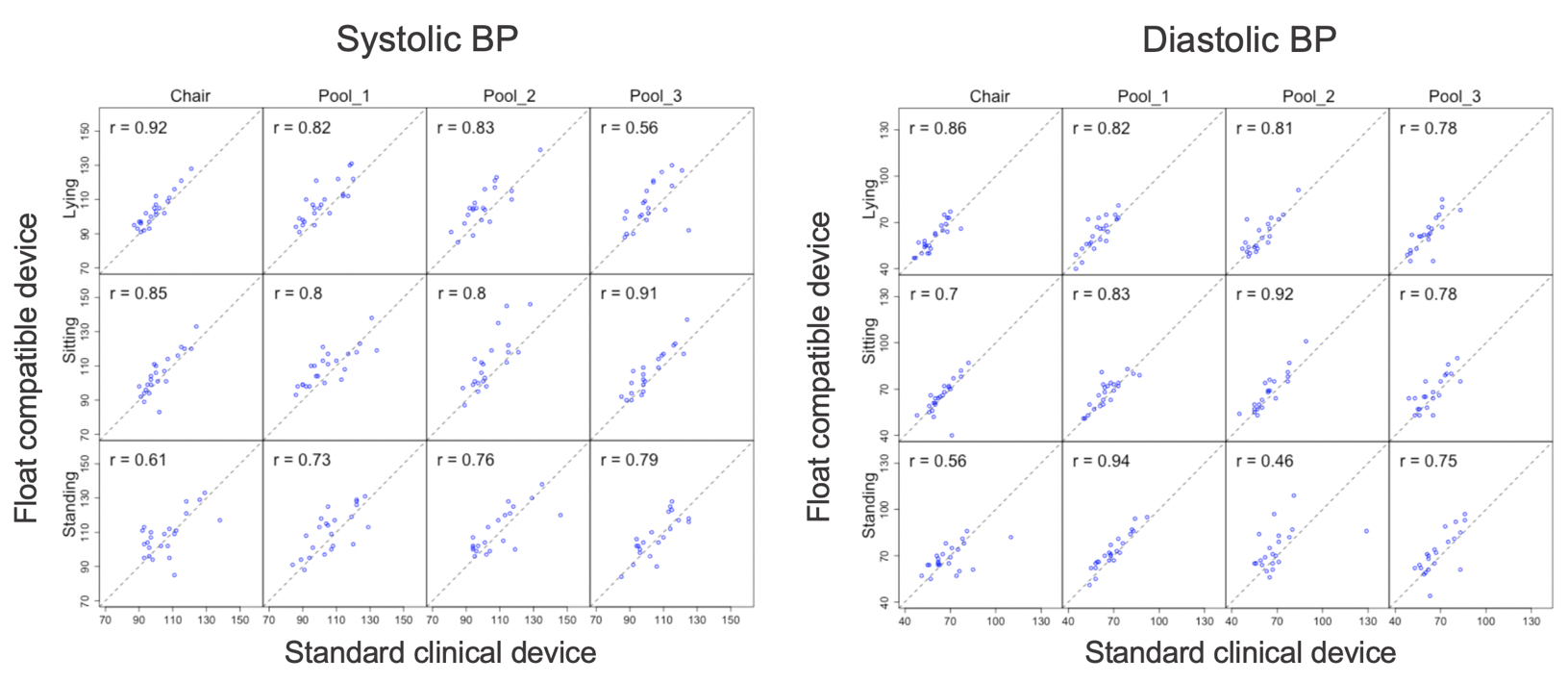
**

**Supplemental Figure 2. Reliability check for blood pressure (BP) devices.** Reliability was assessed for each visit by computing the intraclass correlation coefficient (ICC) between the standard clinical device (CASMED 740) and the float compatible device (QardioArm wireless BP monitor secured in a waterproof sleeve). The overall reliability for the systolic BP measurements was 0.70, and for the diastolic BP measurements was 0.73. All units are in millimeters of mercury (mmHg).
